# Clinical outcomes of on-pump versus off-pump coronary-artery bypass surgery: a meta-analysis

**DOI:** 10.1097/JS9.0000000000001481

**Published:** 2024-04-16

**Authors:** Liaoming He, Xieraili Tiemuerniyazi, Lianxin Chen, Ziang Yang, Shengkang Huang, Yifeng Nan, Yangwu Song, Wei Feng

**Affiliations:** Fuwai Hospital, National Center for Cardiovascular Diseases, Chinese Academy of Medical Sciences and Peking Union Medical College, Beijing, People’s Republic of China

**Keywords:** coronary artery graft bypassing, mortality, off-pump, on-pump, stroke

## Abstract

**Background:**

The ongoing debate regarding off-pump coronary artery bypass grafting (CABG) and on-pump CABG has endured for over three decades. Although numerous randomized controlled trials (RCTs) and meta-analyses have been reported, new evidence has emerged. Therefore, an updated and comprehensive meta-analysis to guide clinical practice is essential.

**Materials and Methods:**

A comprehensive search for eligible articles published after 2000, reporting RCTs involving at least 100 patients and comparing off-pump CABG with on-pump CABG, was performed throughout the databases including Embase, Ovid Medline, and Web of Science. The primary interested outcomes included the short-term incidence of stroke and long-term mortality. The primary analysis utilized fixed-effect model with the inverse variance method. The Grade of Recommendations Assessment, Development, and Evaluation (GRADE) was used to evaluate the certainty of evidence.

**Results:**

After thorough screening, 39 articles were included, consisting of 28 RCTs and involving a total of 16 090 patients. Off-pump CABG significantly reduced the incidence of short-term stroke (1.27 vs. 1.78%, OR: 0.74, *P*=0.03, high certainty). However, it was observed to be associated with increased mid-term coronary reintervention (2.77 vs. 1.85%, RR: 1.49, *P*<0.01, high certainty) and long-term mortality (21.8 vs. 21.0%, RR: 1.09, *P*=0.02, moderate certainty).

**Conclusions:**

Off-pump CABG significantly reduces the short-term incidence of stroke, but it also increases the incidence of mid-term coronary reintervention. Moreover, it may increase long-term mortality.

## Graphical abstract

**Figure:**
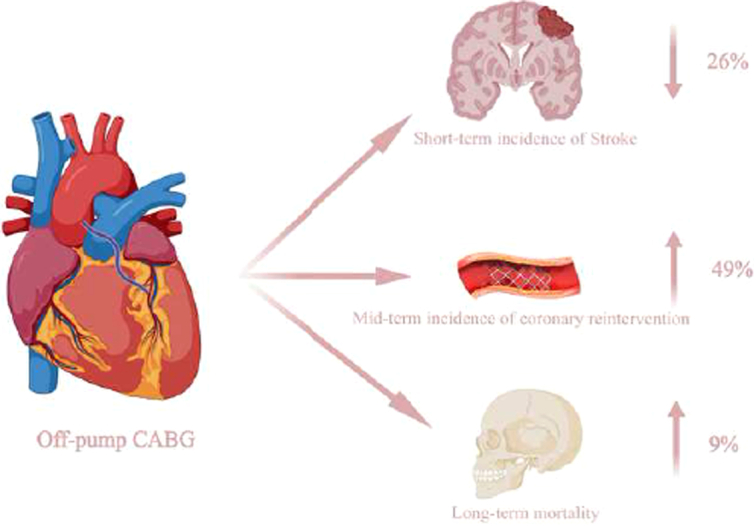

## Introduction

Highlights

- The debates regarding the clinical outcomes of on-pump and off-pump coronary artery bypass grafting (CABG) have persisted for more than three decades.
- In this updated meta-analysis, multiple clinical outcomes on different timepoints after on-pump and off-pump CABG were compared.
- Off-pump CABG significantly reduces the short-term incidence of stroke, but it also increases the incidence of mid-term coronary reintervention. Moreover, it may increase long-term mortality.
- Off-pump CABG may be performed by experienced cardiac surgeons to maximize its clinical benefit.

The debates regarding the clinical outcomes of on-pump and off-pump coronary artery bypass grafting (CABG) have persisted for more than three decades^1^. Theoretically, on-pump CABG offers a surgical field free of motion and blood, facilitating the safe construction of proximal and distal anastomoses. However, the usage of cardiopulmonary bypass is considered to increase perioperative morbidity, such as stroke caused by the manipulation on the ascending aorta^2^. Due to the avoidance of the usage of cardiopulmonary bypass, off-pump CABG has aroused the interest of cardiac surgeons. Several studies have demonstrated off-pump CABG was associated with reduced risk of early mortality, perioperative neurological events, and acute kidney injury (AKI)^3–6^. However, off-pump CABG showed higher rate of incomplete revascularization, increasing the risk of repeat revascularization, and thus potentially increasing the long-term mortality^6–8^.

While numerous meta-analyses regarding the comparison of these two techniques have been reported, they mostly focus on a single clinical outcome. Additionally, it is necessary to further summarize the results of newest evidence. This study is aimed to integrate the high-quality evidence from the randomized controlled trials (RCTs) comparing the clinical outcomes after on-pump and off-pump CABG, thus providing a comprehensive comparison to these two techniques.

## Material and methods

This systematic review and meta-analysis was reported in line with PRISMA (Preferred Reporting Items for Systematic Reviews and Meta-Analyses) and AMSTAR (Assessing the methodological quality of systematic reviews) Guidelines^9,10^. The study has been registered at the International Prospective Register of Systematic Reviews (PROSPERO).

### Search strategy

To identify potential articles, systematic searches were carried out using the following database: Embase, Ovid Medline, and Web of Science. The search was supplemented by scanning the reference lists of eligible articles. A comprehensive account of the literature search strategy can be found in Table S1 (Supplemental Digital Content 1, http://links.lww.com/JS9/C402) along with Figure S1 (Supplemental Digital Content 1, http://links.lww.com/JS9/C402). All identified articles were independently evaluated by two reviewers, with a third reviewer consulted to mediate any disagreements. Searches of published articles were conducted up until 10 July 2023.

### Type of studies to be included

Only studies with an RCT design enrolling a minimum of 100 patients and comparing the clinical outcomes of on-pump and off-pump surgery, and published after the year of 2000 were screened for eligibility for inclusion in this meta-analysis. Language restrictions were not applied. Studies other than RCT, such as animal studies, observational studies, reviews, conference abstracts, case reports, and letters, were excluded. Studies without the desired outcome measures were also excluded. In cases where multiple publications emerged from the same trial, only the primary study was considered, while the others used for subgroup analysis or post-hoc analysis excluded.

### Outcomes

The results were divided into three distinct time periods: short-term (during hospitalization or within 30 days), mid-term (1 year after the surgery), and long-term (more than 4 year after the surgery). The primary interested outcomes were the incidence of stroke in the short-term and all-cause mortality in the long-term. The secondary interested outcomes included stroke and all-cause mortality in the time points other than those in the primary interested outcomes, as well as the incidence of AKI in the short-term and coronary reintervention in the different time periods.

### Data extraction, quality assessment, and variable definition

Two reviewers independently extracted and collected the data from the included studies. Any disagreement between the two reviewers were discussed until they reached a consensus, consulting a third reviewer when necessary. The following data were extracted: trial demographics (the first author, publication year, period during which trials were performed, number of centers, number of subjects randomized and included in analysis, crossover rate), baseline characteristics of the patients (age, sex, diabetes mellitus, hypertension, prior myocardial infarction, prior cerebrovascular accident (CVA), left ventricular ejection fraction), and the clinical outcomes. Hazard ratio (HR) with its 95% CI for survival outcomes were also collected.

### Statistical analysis

#### Risk of bias assessment

The quality of each study was assessed based on the Cochrane Collaboration’s tool for assessing risk of bias. When the number of studies in the meta-analysis exceeded 10, publication bias was assessed by using funnel plots and Begg’s test. Begg’s test, an adjusted rank correlation test, was proposed as technique for quantificationally assess publication bias in a meta-analysis^11^. A significant result from the test indicated the presence of publication bias.

#### Strategy for data synthesis

Fixed-effect model with the inverse variance method was applied in the primary analysis. Mean difference was used for comparison of mean number of grafts. Odds ratio (OR) was used for short-term outcome, while risk ratio (RR) was used for mid-term and long-term outcome. Moreover, HR along with its 95% CI was also employed to assess long-term survival outcome. In cases where studies reported long-term survival outcomes but did not provide the HR, the HR along with its 95% CI were derived using their survival curves through Engauge Digitizer 4.1^12^. Firstly, the origin and axes of the coordinate system were established based on the original survival curve, thereby constructing a two-dimensional coordinate system. Subsequently, the coordinates of any point along the curve were determined using this coordinate system. Intermediate data was then obtained by sampling points along the survival curve. Finally, the intermediate data was transformed into log-rank observed minus expected events and log-rank variance by the method proposed by Tierny *et al*.^13^, enabling a meta-analysis of hazard ratio to be conducted. All meta-analyses were presented with forest plots. The heterogeneity among studies was evaluated by the *χ* ^2^ test and calculated using the *I* ^2^ statistic.

#### Sensitivity analyses

To test the robustness and consistency of the results, random-effect model with the inverse variance effect and leave-one-out analyses were performed. Furthermore, the evaluation of long-term mortality was exclusively based on studies that provided 5-year mortality rates, with the objective of reducing the impact of varying follow-up durations on the mortality. Additionally, the assessment will focus solely on studies that directly presented HR with their 95% CIs, aiming to mitigate the potential influence of HR and its corresponding 95% CI, extracted from survival curves, on the final results. Lastly, univariable meta-regression analyses were performed to determine whether the effects of off-pump CABG were modulated by various factors, including age, sex, diabetes mellitus, difference in the number of grafts, and crossover rate. The difference in the number of grafts was calculated by the differences between the mean number of grafts performed in the off-pump and the on-pump CABG within each trial. The crossover rate served as an indicator of the surgeon’s experience within each trial, encompassing not only the crossover from off-pump CABG to on-pump CABG but also the reverse. Additionally, an analysis examining the occurrence of stroke in relation to prior CVA was performed. If a significant modulated effect was observed in any of these factors, subgroup analyses would be conducted accordingly.

#### Trial sequential analysis

Cumulative meta-analyses are at the risk of producing random errors because of repetitive testing on accumulating data. To minimize random errors, we calculated the required information size (i.e. the number of participants needed in a meta-analysis to detect or reject a certain intervention effect). Information size was based on the assumption of a plausible RR reduction 25%. Our default settings included a type 1 error of 5%, a type 2 error of 20%, and adjustments for heterogeneity in the data. The incidence of the outcomes of interest in the control group was calculated as the median of the incidence of those outcomes in on-pump CABG within each trial, with trials where no events occurred being excluded.

The statistical analysis was conducted using the RevMan software (version 5.4.1, Cochrane Collaboration), STATA (version 13.0, StataCorp), Engauge Digitizer 4.1 (developed by Mark Mitchell, Baurzhan Muftakhidinov, Tobias Winchen, *et al*. Webpage: http://markummitchell.github.io/engauge-digitizer) and Trial Sequential Analysis (TSA) [Computer program]. (Version 0.9.5.10 Beta. The Copenhagen Trial Unit, Centre for Clinical Intervention Research, The Capital Region, Copenhagen University Hospital).

### Certainty assessment

The certainty of evidence was graded by using the Grade of Recommendations Assessment, Development, and Evaluation (GRADE). This assessment was carried out with the assistance of GRADEpro GDT. (GRADEpro GDT: GRADEpro Guideline Development Tool [Software]. McMaster University and Evidence Prime, 2022. Available from gradepro.org.) The principles of GRADE used in this article are elaborated in Table S2.

## Results

### Study characteristic

The primary literature search yielded 406 articles spanning from 2000 to 2023, and 39 articles comprised of 28 RCTs (8 of which were multicenter) were confirmed for eligibility in the final analysis^14–52^. One of the RCTs had three parallel arms, and we specifically extracted the data regarding on-pump and off-pump CABG^27^. The operative crossover rate for off-pump CABG (i.e. patients initially randomized for off-pump CABG but ultimately underwent on-pump CABG) varied from 0 to 15%, whereas it ranged from 0 to 7.9% for on-pump CABG. The duration of follow-up ranged from 1 month to 10 years, while several of the follow-up was limited to intrahospital record (Table S3).

This meta-analysis comprised of 16 090 patients, and the sample size of the included RCTs ranged from 103 to 4752. Most of the patients in these RCTs were at 60–70 years of age, while two RCTs reported patients with a mean age below 60, and 8 reported a mean age above 70. The distribution of baseline characteristics in these RCTs was generally balanced. Among all patients, 21.2% were females, 36.2% had diabetes mellitus, 71.7% had hypertension, 39.8% had prior myocardial infarction, 8.8% had prior CVA and 31.7% had a left ventricular ejection fraction less than 50% (Table S4). The mean number of grafts performed per patient for off-pump CABG ranged from 1.74 to 3.84, while it ranged from 1.80 to 3.75 in on-pump patients. Overall, the mean number of grafts performed in off-pump CABG was lower than on-pump CABG (Table S5 and Figure S2).

### Primary interested outcomes

Twenty-four RCTs reported the incidence of short-term stroke. Overall, 95 (1.27%) out of 7468 patients experienced short-term stroke in off-pump CABG group, whereas 133 (1.78%) out of 7485 suffered from stroke among on-pump CABG patients (OR: 0.74, 95% CI: 0.57–0.97, *P*=0.03) (Figure S3.1).

**Figure 1:**
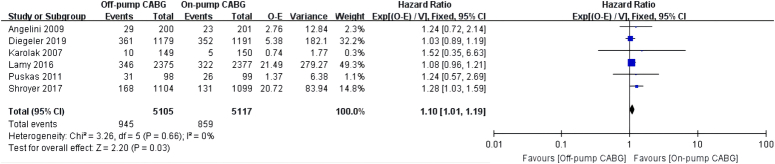
Long-term all-cause mortality. The meta-analysis of long-term all-cause mortality was based fixed-effect method and hazard ratio (HR) value. The HR values, along with their corresponding 95% CI, were calculated based on the survival curves of Karolak 2007 and Puskas. The remaining HR values and their 95% CI were directly extracted from relevant studies.

Eight RCTs reported the incidence of long-term mortality. Overall, 21.8% (1178/5402) of the patients died in the off-pump CABG group, while 20.0% (1084/5409) of the patients died in the on-pump CABG group (RR: 1.09, 95% CI: 1.01–1.17, *P*=0.02) (Figure S3.2). Six articles reported the hazard ratio of long-term mortality (HR: 1.10, 95% CI: 1.01–1.19, *P*=0.03) (Fig. 1).

### Secondary interested outcomes

The incidence of mid-term and long-term stroke were comparable between off-pump CABG (2.03 and 2.43%) and on-pump CABG (2.61 and 2.85%) (mid-term RR: 0.79, 95% CI: 0.60–1.05, *P*=0.10; long-term RR: 0.79, 95% CI: 0.57–1.11, *P*=0.17) (Figure S3.3 and Figure S3.4).

Short-term and mid-term mortality were reported in 25 and 11 of the RCTs, respectively. The short-term and mid-term mortality were comparable between the off-pump (1.90 and 5.12%, respectively) and on-pump CABG (2.24 and 5.03%, respectively) groups (short-term OR: 0.89, 95% CI: 0.71–1.12, *P*=0.32; long-term RR: 1.02, 95% CI: 0.87–1.20, *P*=0.79) (Figure S3.5 and S3.6, respectively).

The number of studies reporting the incidence of coronary reintervention in the short-term, mid-term, and long-term were 7, 9, and 6, respectively. The incidence of coronary reintervention during these time periods were 0.84, 2.77, and 7.25% in the off-pump patients, respectively, which were 0.34, 1.85, and 6.42% in the on-pump patients, respectively (short-term OR: 2.40, 95% CI: 1.26–4.59, *P*=0.01; mid-term RR: 1.49, 95% CI: 1.16–1.92, *P*<0.01; long-term RR: 1.11, 95% CI: 0.96–1.28, *P*=0.15) (Figure S3.7, S3.8, and Figure S3.9, respectively).

Ten RCTs reported the incidence of short-term acute renal failure requiring dialysis. Overall, the rate was 1.4% (81/5773) in the off-pump CABG group, while it was 1.7% (96/5772) among the on-pump CABG patients (OR: 0.85, 95% CI: 0.63–1.15, *P*=0.29) (Figure S3.10).

### Sensitivity analysis

The results for these outcomes were consistent in the sensitivity analysis of using random-effect model and leave-one-out analyses (Table S6, Figure S4, and Figure S5). In the case of long-term mortality, the sensitivity analysis, which conducted exclusively using studies that presented 5-year mortality rates or solely using studies that directly reported the HR along with its corresponding 95% CI, were also consistent with the main analysis of long-term mortality (Figure S6.1 and Figure S6.2).

There was a significant modulated effect for crossover rate on short-term mortality and short-term coronary reintervention. However, no significant modulated effect was observed for the remaining variables on other outcomes (Table S7). Additionally, the modulated effect of prior CVA on short-term stroke was also not significant. Subgroup analysis based on the crossover rate within each trial showed that in trials with a crossover rate <5%, off-pump CABG significantly reduced short-term mortality, while it did not increase short-term coronary reintervention (Figure S7.1 and Figure S7.2).

### Trial sequential analysis

When the information size was based on the assumption of a plausible RR reduction 25%, it was firmly established that off-pump reduced the incidence of short-term stroke while increasing the incidence of mid-term coronary reintervention (Figure S8.2 and Figure S8.7). Additionally, it was firmly established that there was no significant difference in short-term and mid-term mortality between off-pump CABG and on-pump CABG (Figure S8.1 and Figure S8.5). However, the evidence regarding long-term mortality was not as robust as the former findings, suggesting that off-pump CABG may potentially increase long-term mortality compared to on-pump CABG (Figure S8.8). For long-term mortality and other outcomes, further trials are necessary to ascertain conclusive evidence (Table S8 and Figure S8).

### Risk of bias assessment

One of the RCTs was evaluated to have a high risk of random sequence generation and allocation concealment, while another one with a high risk of incomplete outcome data. Additionally, the risk of random sequence generation was unclear in two RCTs, and the risk of allocation concealment was unclear in seven RCTs (Table S9 and Figure S9). Overall, the quality of these RCTs was considered high. For the publication bias, all funnel plot exhibited symmetry (Figure S10, Supplemental Digital Content 1, http://links.lww.com/JS9/C402), and the results of Begg’ test were more than 0.05 (Table S10, Supplemental Digital Content 1, http://links.lww.com/JS9/C402).

### Certainty of evidence

**Figure 2:**
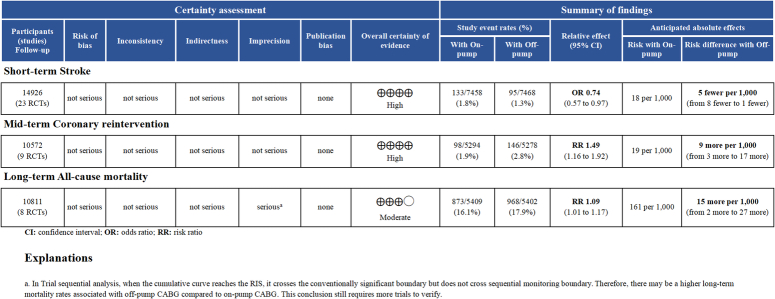
Certainty of evidence of primary interested endpoint.

Figure 2 presents the certainty of evidence for primary interested outcomes, while the certainty of evidence for the secondary outcomes is elaborated in Figure S11 (Supplemental Digital Content 1, http://links.lww.com/JS9/C402). The evidence strongly suggested that off-pump CABG can lower the incidence of short-term stroke and increase the incidence of mid-term coronary reintervention. However, the certainty was moderate regarding the higher long-term mortality associated with off-pump CABG.

## Discussion

To our knowledge, this is the largest and the most comprehensive meta-analysis of RCTs comparing the clinical outcomes of off-pump and on-pump CABG till now, in which the GRADE was applied to grade the certainty of evidence. This meta-analysis included 39 articles, incorporates data from 29 RCTs and involved 16 090 patients. The results of this meta-analysis, basing on high certainty of evidence, suggested that off-pump CABG can reduce the incidence of short-term stroke but increase the incidence of mid-term coronary reintervention. Moreover, with moderate certainty of evidence, off-pump CABG may be associated with higher long-term mortality.

The reduced incidence of short-term stroke after off-pump CABG can be attributed to a decrease in manipulation of the ascending aorta. On-pump CABG involves at least four procedures associated with the ascending aorta, including proximal graft anastomosis, aortic cross-clamping, cannulation for the return of oxygenated blood and cannulation for cardioplegia. Previous meta-analysis and large propensity-matched observational studies also have shown that off-pump CABG reduces the incidence of short-term stroke compared with on-pump CABG^3,4,53^. Notably, this reduction primarily pertains to short-term stroke of embolic origin, rather than a reduction in delayed strokes due to various causes^54^. This connection is further supported by a network meta-analysis, which demonstrated that anaortic off-pump CABG has a lower incidence of stroke compared to off-pump CABG with partial clamp^55^. In this study, the short-term incidence of stroke is in line with the previous studies, showing lower incidence in off-pump patients. Additionally, even though the mid-term and long-term rates of stroke did not reach statistical significance, the point estimation in meta-analysis and the cumulative curve in trial sequential analysis tended to favor off-pump CABG. This trend might also be attributed to the reduction of short-term stroke rates.

Another notable finding of this study was that, compared with on-pump CABG, there was a high certainty of evidence indicating that off-pump CBAG increased the incidence of mid-term coronary reintervention. The increased incidence of mid-term coronary reintervention after off-pump CABG should be attributed to incomplete coronary revascularization and increased incidence of venous grafts to occlusion^56^. In this study, the mean grafts in off-pump CABG were significantly fewer in off-pump CABG patients, while the meta-regression indicated that the difference in the number of grafts did not have a modulated effect on coronary reintervention. Despite that, it cannot be excluded that incomplete coronary reintervention may contributed to more coronary reintervention in off-pump CABG. This consideration aroused because the analysis was based on the trial baseline rather than the individual baseline. However, the meta-regression suggested that the crossover rate had a modulated effect on short-term coronary reintervention. Subgroup analysis implied surgeon’s surgical expertise in off-pump CABG may reduce the incidence of short-term mortality.

Another crucial finding of this study was that, compared with on-pump CABG, there was a moderate certainty of evidence indicating long-term mortality of off-pump CABG was significantly increased. Earlier meta-analyses and large propensity-matched observational studies have indicated a significant increase in long-term mortality associated with off-pump CABG^6,8,53,57,58^. The increased incidence of long-term mortality in off-pump CABG is logically reasonable, given the increased incidence of mid-term coronary reintervention. In this meta-analysis, both primary analysis and sensitivity analysis consistently demonstrated that off-pump significantly increased long-term mortality. However, the trial sequential analysis indicated the definitive conclusion should be approached with cautious. Additionally, the 10-year follow-up of the ROOBY trial does not indicate a significant increase in long-term mortality with off-pump CABG compared to on-pump CABG^50^. This is contrasts with the 5-year follow-up, which shows a significant increase in long-term mortality with off-pump CABG^52^. As a result, we have decided to downgrade the certainty of evidence regarding the increased long-term mortality associate with off-pump CABG because of imprecision bias. No significant difference was observed regarding the short-term and mid-term mortality. Interestingly, the point estimate for the short-term mortality tended to favor off-pump CABG. Subgroup analysis even indicated that for crossover rate less than 5%, off-pump CABG can significantly reduce the short-term mortality. The possible explanation is that off-pump CABG reduced the short-term mortality through decreasing the incidence of stroke^2^. For the mid-term mortality, the point estimate was neutral. This implicated that the benefit of reducing the incidence of short-term stroke after off-pump CABG might be completely offset by the increased incidence of mid-term coronary reintervention.

There was no significant difference in the incidence of acute renal failure requiring dialysis. In theory, the use of cardiopulmonary bypass can trigger a systematic inflammatory response and elevate free hemoglobin levels due to ruptured red blood cells, both of which contribute to an increase risk of AKI. However, in off-pump CABG, hemodynamic instability due to the manipulation on the heart during the anastomoses between graft and posterolateral vessels, might also impair renal perfusion and lead to AKI. Therefore, the results were neutral. Our finding is also consistent with prior meta-analyses, reporting that off-pump CABG is associated with a lower incidence of postoperative AKI but does not increase the need for dialysis^5,59^.

### Strength and limitations

Although there have been a number of meta-analyses on this topic, our review has own strengths. First, we have employed specific inclusion criteria for the studies included in this meta-analysis to minimize between-trial variance. On the one hand, we included trials reporting a minimum of 100 patients to reduce the small sample size effect. On the other hand, only studies published after the year of 2000 were included, considering the improvements of surgical techniques. Second, compared to prior meta-analyses which focused solely on the overall effect regarding a single endpoint (Table S12, Supplemental Digital Content 1, http://links.lww.com/JS9/C402), this meta-analysis investigated multiple clinical endpoints across various time points, which offer a comprehensive, robust, and accurate comparison between on-pump and off-pump CABG. Last but by no means the least, we employed GRADE to assess the studies included, clearly presenting the certainty of evidence.

Our analysis has several significant limitations. Firstly, our findings and interpretations are based on trial-level rather than individual-level data. Consequently, our conclusions are limited by the characteristics and methodologies of the included trials themselves. Secondly, the meta-regression technique is based on trial-level characteristics. Therefore, its finding should be regarded as hypothesis-generating rather than definite conclusions. Additionally, it would have been intriguing to assess the modulated effects of additional factors such as renal function, pulmonary disease, surgical risk score, and aortic atheroma. However, these variables were not consistently reported in the studies. Lastly, despite the inclusion of 28 RCTs in this meta-analysis, only 8 RCTs reported their long-term outcomes, thus limiting the certainty of evidence for these outcomes.

## Conclusion

**Figure 3:**
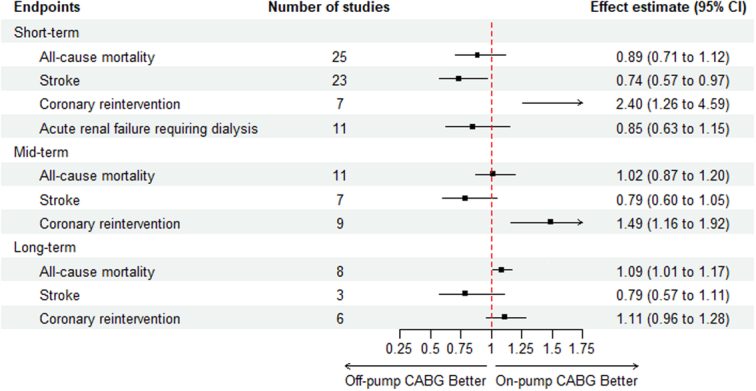
Forest plot for all endpoints. For short-term outcomes, effect estimate denotes odds ratio (OR), while for mid-term and long-term outcomes, effect estimate denotes risk ratio (RR). CABG, coronary artery bypass grafting.

Off-pump CABG significantly reduces the short-term incidence of stroke, but it also increases the incidence of mid-term coronary reintervention. Moreover, it may increase long-term mortality (Fig. 3).

## Ethical approval

Not applicable.

## Consent

Not applicable.

## Sources of funding

This work was supported by the National High Level Hospital Clinical Research Funding (No. 2022-GSP-GG-30) and the National Key Research and Development Program [2018YFC1311201] from the Ministry of Science and Technology of the People’s Republic of China.

## Author contribution

L.H. and X.T.: design the work and finished the writing of the original draft together, and contributed equally to the study; L.C. and Z.Y.: participated in the collection of data; Y.N., S.H., and Y.S.: analysis and interpretation of data; W.F.: participated in study design, planning, and carrying out, provided expert guidance to analysis and writing up, and administered and supervised this work.

## Conflicts of interest disclosure

All authors declare no conflict of interest.

## Research registration unique identifying number (UIN)

This systematic review and meta-analysis was reported following the Preferred Reporting Items for Systematic reviews and Meta-analyses (PRISMA) guidelines. The study has been registered at the International Prospective Register of Systematic Reviews (PROSPERO, No. CRD42023443009).

## Guarantor

All authors accept full responsibility for the work and the conduct of the study, had access to the data, and controlled the decision to publish.

## Data availability statement

The data utilized in this meta-analysis all exclusively derived from published articles.

## Provenance and peer review

Not commissioned, externally peer-reviewed.

## Supplementary Material
