# Supplementary material for: Clinical outcomes of on-pump versus off-pump coronary-artery bypass surgery: a meta-analysis
Source: Int J Surg. 2024 Apr 16;110(8):5063–70. doi: 10.1097/JS9.0000000000001481 (PMC11325898; doi:10.1097/JS9.0000000000001481)
Supplement: Supplementary file 1 [file js9-110-5063-s001.pdf]

On-Pump versus Off-Pump Coronary-Artery Bypass Surgery: A meta-analysis

-------Supplement material

Table S1. Literature search strategy 1
Table S2. Principles of Grade of Recommendations Assessment, Development, and Evaluation (GRADE) 2
Table S3 The trial demographic of trials included in this meta-analysis 3
Table S4. Patient baseline characteristics of trials included in this meta-analysis 5
Table S5. Number of grafts performed per patient 7
Table S6. The outcomes of all meta-analyses 9
Table S7. The outcomes of meta-regression 10
Table S8. The outcomes of Trial sequential analysis 11
Table S9. The Risk of bias in randomized controlled trials 12
Table S10. The outcomes of Begg's test 14
Table S11. Outcome definition 15
Table S12. Prior meta-analysis which only included randomized controlled trials 18
Figure S1. Flow of diagram 20
Figure S2. Forest plot of number of anastomoses/grafts per patient 21
Figure S2.1 No of anastomoses/grafts per patient (Fixed effect) 21
Figure S2.2 No of anastomoses/grafts per patient (Random effect) 21
Figure S3. Forest plot of clinical outcome (Fixed effect) 22
Figure S3.1 Short-term Stroke 22
Figure S3.2 Long-term Mortality 22
Figure S3.3 Mid-term Stroke 23
Figure S3.4 Long-term Stroke 23
Figure S3.5 Short-term Mortality 24
Figure S3.6 Mid-term Mortality 24
Figure S3.7 Short-term Coronary reintervention 25
Figure S3.8 Mid-term Coronary reintervention 25
Figure S3.9 Long-term Coronary reintervention 25
Figure S3.10 Short-term Acute renal failure requiring dialysis 26
Figure S4. Forest plot of clinical outcome (Random effect) 27
Figure S4.1 Short-term Stroke 27
Figure S4.2 Long-term Mortality 27
Figure S4.3 Mid-term Stroke 28
Figure S4.4 Long-term Stroke 28
Figure S4.5 Short-term Mortality 29
Figure S4.6 Mid-term Mortality 29
Figure S4.7 Short-term Coronary reintervention 30
Figure S4.8 Mid-term Coronary reintervention 30
Figure S4.9 Long-term Coronary reintervention 30
Figure S4.10 Short-term Acute renal failure requiring dialysis 31
Figure S5. Leave-one-out analysis 32
Figure S5.1 Short-term stroke 32
Figure S5.2 Long-term mortality 32
Figure S5.3 Mid-term stroke 32
Figure S5.4 Long-term stroke 33
Figure S5.5 Short-term mortality 33
Figure S5.6 Mid-term mortality 33
Figure S5.7 Short-term coronary reintervention 34
Figure S5.8 Mid-term coronary reintervention 34
Figure S5.9 Long-term coronary reintervention 34
Figure S5.10 Short-term acute renal failure requiring dialysis 35
Figure S6. Sensitivity analysis 36

Figure S6.1 Mortality based on studies reporting 5-year Mortality.....36
Figure S6.2 Mortality based on studies directly reporting HR.....36
Figure S7. Subgroup analysis based on crossover rate.....37
Figure S7.1 Subgroup analysis for short-term mortality.....37
Figure S7.2 Subgroup analysis for short-term coronary reintervention.....37
Figure S8. Trial sequential analysis.....38
Figure S8.1 Short-term mortality.....38
Figure S8.2 Short-term stroke.....39
Figure S8.3 Short-term coronary reintervention.....40
Figure S8.5 Mid-term mortality.....42
Figure S8.6 Mid-term stroke.....43
Figure S8.7 Mid-term coronary reintervention.....44
Figure S8.8 Long-term mortality.....45
Figure S8.9 Long-term stroke.....46
Figure S8.10 Long-term coronary reintervention.....47
Figure S9. Study-specific risk of bias.....48
Figure S9.1 Risk of bias graph in study of report short-term outcome.....48
Figure S9.2 Risk of bias summary in study of report short-term outcome.....48
Figure S9.3 Risk of bias graph in study of report mid-term outcome.....49
Figure S9.4 Risk of bias summary in study of report mid-term outcome.....49
Figure S9.5 Risk of bias graph in study of report long-term outcome.....50
Figure S9.6 Risk of bias summary in study of report long-term outcome.....50
Figure S10. Funnel plot.....51
Figure S10.1 Funnel plot in short-term mortality.....51
Figure S10.2 Funnel plot in short-term stroke.....51
Figure S10.3 Funnel plot in short-term acute renal failure requiring dialysis.....51
Figure S10.4 Funnel plot in mid-term Mortality.....51
Figure S11. GRADE evidence of profile and summary of Findings table.....52
Figure S11.1 GRADE evidence of profile of short-term outcomes.....52
Figure S11.2 GRADE evidence of profile of mid-term outcomes.....53
Figure S11.3 GRADE evidence of profile of long-term outcomes.....54

Table S1. Literature search strategy

EMBASE:

#1	'off pump':ab,ti OR 'off-pump':ab,ti OR 'without cardiopulmonary bypass':ab,ti OR 'beating heart':ab,ti
#2	'on pump':ab,ti OR 'on-pump':ab,ti OR 'with cardiopulmonary bypass':ab,ti OR 'cardioplegic arrest':ab,ti
#3	'coronary artery bypass':ab,ti OR 'coronary artery bypass graft':ab,ti OR 'coronary artery bypass grafting':ab,ti OR 'coronary artery bypass surgery':ab,ti OR 'coronary artery surgery':ab,ti
#4	'coronary bypass':ab,ti OR 'coronary bypass graft':ab,ti OR 'coronary bypass grafting':ab,ti OR 'coronary bypass surgery':ab,ti OR 'coronary surgery':ab,ti
#5	'randomized controlled trial'/exp
#6	'randomised controlled study':ab,ti OR 'randomised controlled trial':ab,ti OR 'randomized controlled study':ab,ti OR 'randomized controlled trial':ab,ti
#7	#1 AND #2
#8	#3 OR #4
#9	#5 OR #6
#10	#7 AND #8 AND #9
#13	#7 AND (2000:py OR 2001:py OR 2002:py OR 2003:py OR 2004:py OR 2005:py OR 2006:py OR 2007:py OR 2008:py OR 2009:py OR 2010:py OR 2011:py OR 2012:py OR 2013:py OR 2014:py OR 2015:py OR 2016:py OR 2017:py OR 2018:py OR 2019:py OR 2020:py OR 2021:py OR 2022:py OR 2023:py)

Ovid Medline:

#1	("off pump" or "off-pump" or "without cardiopulmonary bypass" or "beating heart").ab,ti
#2	("on pump" or "on-pump" or "with cardiopulmonary bypass" or "cardioplegic arrest").ab,ti
#3	("coronary artery bypass" or "coronary artery bypass graft" or "coronary artery bypass grafting" or "coronary artery bypass surgery" or "coronary artery surgery").ab,ti
#4	("coronary bypass" or "coronary bypass graft" or "coronary bypass grafting" or "coronary bypass surgery" or "coronary surgery").ab,ti
#5	"randomized controlled trial". pt.
#6	("randomised controlled study" or "randomised controlled trial" or "randomized controlled study" or "randomized controlled trial").ab,ti
#7	#1 AND #2
#8	#3 OR #4
#9	#5 or #6
#10	#7 AND #8 AND #9
#11	limit 10 to yr="2000 -Current"

Web of Science:

#1	TS=("off pump" or "off-pump" or "without cardiopulmonary bypass" or "beating heart")
#2	TS=("on pump" or "on-pump" or "with cardiopulmonary bypass" or "cardioplegic arrest")
#3	(TS=("coronary artery bypass" or "coronary artery bypass graft" or "coronary artery bypass grafting" or "coronary artery bypass surgery" or "coronary artery surgery")) OR TS=("coronary bypass" or "coronary bypass graft" or "coronary bypass grafting" or "coronary bypass surgery" or "coronary surgery")
#4	TS=("randomised controlled study" or "randomised controlled trial" or "randomized controlled study" or "randomized controlled trial")
#5	#1 AND #2
#6	#5 AND #3 AND #4

Table S2. Principles of Grade of Recommendations Assessment, Development, and Evaluation (GRADE)

We employed Gradepro GDT to evaluate the certainty of evidence. This assessment mainly involved five essential aspects: Risk of bias, Inconsistency, Indirectness, Imprecision and Publication bias.

Risk of bias	We employed the Cochrane Collaboration's tool, aiding in the evaluation of the quality of each study that was included (Table S7 and Figure S2)
Inconsistency	The classification of "not serious" is applied only when the point estimate demonstrates minimal variability, the confidence interval exhibits distinct overlap, the heterogeneity test yields a non-significant P value, and the I^2 value is low. The I^2 value could inform the inconsistency of studies. If the I^2 value was below 30%, the level of consistency was considered low. If the I^2 value was between 30% and 60%, the consistency was moderate. When the I^2 value exceeded 60%, the consistency was considered high.
Indirectness	All the studies included in this meta-analysis were randomized controlled trials (RCTs) that directly compared Off-pump CABG with On-pump CABG in patients undergoing their first, isolated CABG procedures. Additionally, each outcome was assessed directly within the original studies, avoiding the use of alternative outcomes. As a result, the inconsistency across all outcomes was considered as "not serious".
Imprecision	The evaluation incorporated the Optimal Information Size (OIS) alongside a 95% Confidence Interval (CI). If the OIS threshold wasn't met, the evaluation of 'Imprecision' would be adjusted downwards. Similarly, if the OIS target was met but the 95% CI encompassed 1.0, the assessment of "Imprecision" would still be downgraded. The determination of the OIS quantity would be grounded in the incidence of outcomes in on-pump CABG procedures and a risk ratio reduction of 25%. This approach closely resembled the required information sizes (RIS) in Trial sequential analysis. Consequently, the RIS took place of the OIS in this context. If the cumulative curve in Trial sequential analysis had reached the RIS, or had crossed a sequential monitoring boundary or an invalid boundary before reaching the RIS (Refer to Table S8 and Figure S8), then the certainty would not be downwards because of "Impression".
Publication bias	Funnel plots and Begg's test were employed to support the assessment when the number of studies exceeded 10. (Refer to Table S9 and Figure S10.) If the count of studies integrated into the meta-analysis was fewer than 10, the existence of "Publication bias" was categorized as "undetected." This methodology was adopted because all the incorporated studies were published after 2000, with the majority being initiated by researchers rather than equipment suppliers.

Table S3 The trial demographic of trials included in this meta-analysis

Trial	Author/Year	Period during which surgeries were performed	Number of centers	Patients included in analysis			Crossover rate, %	
				Total	Off-pump	On-pump	Off-pump	On-pump
BHACAS 1	Angelini 2002<br>Angelini 2009	March 1997 to August 1998	Single-center	200	100	100	2%	0%
BHACAS 2	Angelini 2002<br>Angelini 2009	September 1998 to November 1999	Single-center	201	100	101	0%	0%
	Raja 2003	January 2000 to January 2001	Single-center	300	150	150	NR	NR
	Muneretto 2003	January 2000 to January 2002	Single-center	176	88	88	9%	0%
	Lingaas 2004<br>Lingass 2006	March 1999 to March 2002	Single-center	120	60	60	12%	0%
	Legare 2004<br>Karolak 2007	August 1999 to March 2003	Single-center	300	150	150	13%	1%
PRAGUE-4	Straka 2004	May 2000 to June 2002	Single-center	400	208	192	15%	7%
	Gerola 2004	NR	Multi-center	160	80	80	0%	0%
	Khan 2004	January 2000 to January 2002	Single-center	103	54	49	4%	0%
	Chen 2004	June 2001 to April 2003	Single-center	300	150	150	0%	0%
Octopus	Van Dijk 2001<br>Nathoe 2003<br>Van Dijk 2007	March 1998 to August 2000	Multi-center	281	142	139	7.0%	3.6%
SMART	Puskas 2003<br>Puskas 2004<br>Puskas 2011	March 2000 to August 2001	Single-center	197	98	99	1.0%	3.0%
JOCRI	Kobayashi 2005	July 2002 to September 2004	Multi-center	167	81	86	0.0%	1.2%
	Motallebazadeh 2007	August 2002 to March 2004	Single-center	212	108	104	0.0%	0.0%
	Hernandez 2007	January 2001 to January 2004	Single-center	201	99	102	8.1%	0.0%
	Nogueira 2008	January 2002 and December 2006	Single-center	202	105	97	NR	NR
	Naseri 2009	September 2007 to March 2008	Single-center	120	60	60	NR	NR

ROOBY	Shroyer 2009<br>Shroyer 2017<br>Quin 2022	February 2002 to May 2008	Multi-center	2203	1104	1099	12.4%	3.6%
	Iqbal 2014	January 2006 to March 2007	Single-center	200	100	100	0.0%	1.0%
PROMISS	Sousa Uva 2010	April 2005 to July 2007	Single-center	147	73	74	1.3%	2.7%
MASS III	Hueb 2010	March 2001 to March 2006	Single-center	308	155	153	1.9%	0.0%
BBS	Møller 2010	April 2002 to March 2006	Single-center	339	176	163	4.5%	3.7%
on-off	Lemma 2012	December 2006 to April 2010	Multi-center	411	208	203	3.80%	7.90%
DOORS	Houlind 2012	January 2005 to November 2008	Multi-center	900	450	450	13.3%	2.6%
CORONARY	Lamy 2012<br>Lamy 2013<br>Lamy 2016	November 2006 to October 2011	Multi-center	4752	2375	2377	7.9%	6.4%
	Lei 2014	January 2011 to October 2013	Single-center	445	222	223	0.0%	2.2%
PRAGUE-6	Hlavicka 2016	June 2006 to July 2011	Single-center	206	98	108	8.5%	0.0%
GOPCABE	Diegeler 2013<br>Diegeler 2019	June 2008 to september 2011	Multi-center	2539	1271	1268	9.7%	5.1%

NR denotes not reported

Table S4. Patient baseline characteristics of trials included in this meta-analysis

Author/Year	Age, years		Female, %		DM, %		Hypertension, %		Prior MI, %		Prior CVA, %		LVEF<50%, %	
Angelini 2002 Angelini 2009	62.2 (9.6)	61.7 (8.6)	18	21	19	14	62	49	41	30	0	0	20	21
Angelini 2002 Angelini 2009	63.8 (8.5)	61.2 (9.2)	18	15	32	30	60	50	50	50	0	0	24	23
Raja 2003	64 (38-66)	64 (45-75)	24.7	24	NR	NR	NR	NR	NR	NR	NR	NR	NR	NR
Muneretto 2003	67 (8)	66 (9)	37.5	41	42	39.7	NR	NR	38.6	40.9	0	0	12.5*	7.9*
Lingaas 2004 Lingass 2006	64 (7.8)	65 (8.3)	13.3	30.0	13.3	20	41.7	43.3	51.7	56.7	8.3	6.7	NR	NR
Legare 2004 Karolak 2007	62.1 (10.1)	63.7 (10.0)	18.7	20.7	29.3	36	70	60	44.7	50.7	8.3	12.0	13.3	15.3
Straka 2004	63	62	23	14	28	29	56	59	58	61	7.0	11.0	22	16
Gerola 2004	59.1 (9.7)	58.9 (8.9)	29	26	19	14	37	43	45	24	NR	NR	NR	NR
Khan 2004	62.0 (7.9)	64.7 (8.4)	7	18	28	26	NR	NR	39	49	4.1	5.6	24	27
Chen 2004	66.5 (8.8)	64.3 (6.2)	26	20	32	28	NR	NR	30.7	26.7	26.0	13.3	NR	NR
Van Dijk 2001 Nathoe 2003 Van Dijk 2007	61.7 (9.2)	60.8 (8.8)	34	29	9	17	40	44	34	26	4	3	23	21
Puskas 2003 Puskas 2004 Puskas 2011	62.2 (11.1)	62.5 (9.45)	22.4	23.2	32.7	33.3	65.3	61.6	34.7	33.3	1.0	9.1	47.7#	48.4#
Kobayashi 2005	60 (7)	59 (10)	13.6	12.8	47	58	67	69	52	47	0	0	NR	NR
Motallebazadeh 2007	63.9 (0.9)	65.1 (0.9)	13	9	19	29	57	66	52	55	0	0	54	51
Hernandez 2007	NR	NR	19.2	20.6	35.4	30.4	NR	NR	NR	NR	NR	NR	17.8	19.1
Nogueira 2008	61	59	30	21	33	27	70	57	44	44	NR	NR	NR	NR
Naseri 2009	71 (6.9)	68 (8.5)	75	76.7	45	50	91.7	98.3	NR	NR	100.0	100.0	NR	NR
Shroyer 2009 Shroyer 2017 Quin 2022	63 (8.5)	62.5 (8.5)	0.6	0.5	42.6	44.7	85.9	86.6	NR	NR	7.4	8.0	40.6#	42#
Iqbal 2014	51.59 (10.30)	53.51 (9.96)	NR	NR	42	38	53	54	NR	NR	0	0	NR	NR

Sousa Uva 2010	66.1 (9.5)	64.6 (9.8)	17.8	14.9	35.6	36.5	83.6	78.4	49.3	55.4	9.6	8.1	7	26
Hueb 2010	61	59	22	20	29	27	34	37	34	32	NR	NR	NR	NR
Møller 2010	76.1 (5.2)	75.6 (4.9)	35	36	18	18	48	53	56[†]	58[†]	17.6	21.5	49	49
Lemma 2012	74	73	29.8	31.5	42.8	43.3	83.7	82.3	69.2	78.3	9.6	9.9	56.8	58.7
Houlind 2012	75 (70-88)	75 (70-91)	24	22	22	18	71	71	44	45	4.5	4.7	34	31
Lamy 2012<br>Lamy 2013<br>Lamy 2016	67.6 (6.7)	67.5 (6.9)	20	19.3	46.5	47.5	76.2	75.5	33.8	35.2	6.7	7.8	29.5	29.1
Lei 2014	64.79 (8.03)	64.94 (8.08)	39.2	37.7	100	100	63.1	65.5	24.3	22	24.8	29.1	NR	NR
Hlavicka 2016	74.7 (6.5)	73.6 (7.4)	40.8	42.6	48	46.3	83.7	83.3	59.2	67.6	12.0	16.3	56.1	52.8
Diegeler 2013<br>Diegeler 2019	78.6 (3.0)	78.4 (2.9)	30.8	32.2	15.1	13.8	NR	NR	36	37.8	10.2	7.9	32.9	31.5

NR denotes not reported, DM denotes diabetes mellitus, MI denotes myocardial infarction, CAV denotes cerebrovascular accident, LVEF denotes left ventricular ejection fraction (LVEF), * denotes LVEF <30%, # denotes LVEF <55%.

Table S5. Number of grafts performed per patient

Trial	Author/Year	One-vessel disease		Two-vessel disease		Three-vessel disease		No. of grafts performed per patient	
BHACAS 1	Angelini 2002<br>Angelini 2009	NR	NR	NR	NR	NR	NR	2.23	2.31
BHACAS 2	Angelini 2002<br>Angelini 2009	NR	NR	NR	NR	NR	NR	2.64	2.89
	Raja 2003	NR	NR	NR	NR	NR	NR	2 (1-4)	2 (1-4)
	Muneretto 2003	9.2	8	42	41	48.8	51	2.7 (0.5)	2.8 (0.8)
	Lingaas 2004<br>Lingass 2006	10.0	11.7	35.0	43.3	55.0	45.0	2.6 (0.9) ⁑	2.8 (1.0) ⁑
	Legare 2004<br>Karolak 2007	6	8	26.7	18	67.3	74	2.8 (0.9)	3.0 (0.9)
PRAGUE-4	Straka 2004	8	4	24	28	68	68	2.3 ⁑	2.7 ⁑
	Gerola 2004	NR	NR	NR	NR	NR	NR	1.74 (0.67)	1.80 (0.66)
	Khan 2004	0	0	0	0	100	100	3.1 (0.6)	3.4 (0.7)
	Chen 2004	0	0	0	0	100	100	3.84 (1.06) ⁑	3.75 (0.94) ⁑
Octopus	Van Dijk 2001<br>Nathoe 2003<br>Van Dijk 2007	30	22	50	50	20	27	2.4 (1.0)	2.6 (1.1)
SMART	Puskas 2003<br>Puskas 2004<br>Puskas 2011	NR	NR	NR	NR	NR	NR	3.39 (1.04)	3.40 (1.08)
JOCRI	Kobayashi 2005	0	0	68	68	32	32	3.5 (1.0)	3.6 (0.9)
	Motallebazadeh 2007	8	2	19	20	72	78	NR	NR
	Hernandez 2007	12.1	15.6	33.3	27.5	54.6	56.9	3.2 (1.0) ⁑	3.3 (0.9) ⁑
	Nogueira 2008	0	0	24.16	26.18	75.84	73.82	NR	NR
	Naseri 2009	NR	NR	NR	NR	NR	NR	NR	NR
ROOBY	Shroyer 2009<br>Shroyer 2017<br>Quin 2022	5.9	6	28.9	26	65.2	68	2.9 (0.9)	3.0 (1.0)

	Iqbal 2014	NR	NR	NR	NR	NR	NR	2.96 (0.942)	2.99 (0.882)
PROMISS	Sousa Uva 2010	0	0	0	0	100	100	3.5 (0.6)	3.5 (0.6)
MASS III	Hueb 2010	0	0	26	24	74	76	2.49	2.97
BBS	Møller 2010	0	0	0	0	100	100	3.22 (0.72)	3.34 (0.76)
on-off	Lemma 2012	NR	NR	NR	NR	NR	NR	3.0 (1.1) ⁑	3.3 (1.0) ⁑
DOORS	Houlind 2012	2	2	17	20	81	78	2.9 (0.9)	3.1 (1.0)
CORONARY	Lamy 2012<br>Lamy 2013<br>Lamy 2016	3	2.1	18.7	16.4	56.1	60.4	3	3.2
	Lei 2014	0	0	0	0	100	100	3.18 (0.64) ⁑	3.22 (0.61) ⁑
PRAGUE-6	Hlavicka 2016	6.1	3.7	19.4	17.6	73.5	78.7	2.04 ⁑	2.66 ⁑
GOPCABE	Diegeler 2013<br>Diegeler 2019	1.9	1.2	10	8.8	60	60.5	2.7	2.8

NR denotes not reported, ⁑ denotes no. of anastomosis per patient

Table S6. The outcomes of all meta-analyses

	Outcome	Studies	Participants	Fixed effect		Random effect	
				Effect estimate (95%CI)	P value	Effect estimate (95%CI)	P value
Short-term	Mortality	25	15610	0.89 (0.71,1.12)	0.32	0.89 (0.71,1.12)	0.32
	Stroke	23	14926	0.74 (0.57,0.97)	0.03	0.74 (0.57,0.97)	0.03
	Coronary reintervention	7	8335	2.40 (1.26,4.59)	0.01	2.24 (1.08,4.64)	0.03
	Acute renal failure requiring dialysis	11	11545	0.85 (0.63,1.15)	0.29	0.85 (0.63,1.15)	0.29
Mid-term	Mortality	11	10950	1.02 (0.87,1.20)	0.79	1.02 (0.87,1.20)	0.79
	Stroke	7	8396	0.79 (0.60,1.05)	0.10	0.79 (0.60,1.05)	0.10
	Coronary reintervention	9	10572	1.49 (1.1.6,1.92)	<0.01	1.49 (1.16,1.92)	<0.01
Long-term	Mortality	8	10811	1.09 (1.01,1.17)	0.02	1.09 (1.01,1.17)	0.02
	Stroke	3	5341	0.79 (0.57,1.11)	0.17	0.79 (0.57,1.11)	0.17
	Coronary reintervention	6	9891	1.11 (0.96,1.28)	0.15	1.11 (0.96,1.28)	0.15

For the short-term outcomes, the effect estimate is represented by the odds ratio (OR), while for the mid-term and long-term outcomes, the effects estimate is represented by the risk ratio (RR).

Table S7. The outcomes of meta-regression

Outcome		Crossover rate	Age	Female	DM	Prior CVA	Difference in the number of grafts
Short-term	mortality	0.020	0.662	0.090	0.873	NA	0.638
	stroke	0.061	0.679	0.118	0.707	0.611	0.074
	coronary reintervention	0.044	0.929	0.368	0.779	NA	0.730
	acute renal failure requiring dialysis	0.496	0.599	0.370	0.808	NA	0.636
Mid-term	mortality	0.662	0.309	0.348	0.159	NA	0.228
	stroke	0.463	0.862	0.596	0.734	0.952	0.576
	coronary intervention	0.874	0.769	0.542	0.839	NA	0.561
Long-term	mortality	0.298	0.275	0.606	0.671	NA	0.539
	stroke	0.531	0.392	0.356	0.327	0.372	0.678
	coronary intervention	0.639	0.512	0.304	0.403	NA	0.746

DM denotes diabetes mellitus, CVA denotes cerebrovascular accident, NA denotes not applied.

Table S8. The outcomes of Trial sequential analysis

		Incidence of interested outcomes	Required information size	Pooled effect	Firm evidence
Short-term	mortality	2.15%	19861	0.85 (0.68,1.06)	√
	stroke	2.63%	16196	0.71 (0.55,0.93)	√
	coronary reintervention	0.93%	70320	2.40 (1.31,4.42)	×
	acute renal failure requiring dialysis	1.16%	37507	0.84 (0.62,1.13)	×
Mid-term	mortality	4.13%	10312	1.02 (0.87, 1.20)	√
	stroke	2.99%	14214	0.78 (0.59,1.03)	×
	coronary reintervention	2.80%	15314	1.49 (1.16,1.92)	√
Long-term	mortality	12.49%	3226	1.06 (0.99,1.04)	×
	stroke	3.27%	12972	0.78 (0.56,1.11)	×
	coronary reintervention	3.46%	12473	1.13 (0.98,1.30)	×

Firm evidence indicated that either the cumulative curve had reached the required information size or had crossed a sequential monitoring boundary or an invalid boundary before reaching the required information sizes in a meta-analysis. The information size was determined assuming a plausible relative risk reduction of 25%, with default setting comprising a type 1 error rate of 5% and a type 2 error rate of 20%.

Table S9. The Risk of bias in randomized controlled trials

Trial	Selection bias		Performance bias	Detection bias	Attrition bias
	Random sequence generation	Allocation concealment	Blinding of participants and personnel	Blinding of outcome assessment	Incomplete outcome data
BHACAS 1	Low risk of bias	Low risk of bias	Low risk of bias	Low risk of bias	Low risk of bias
BHACAS 2	Low risk of bias	Low risk of bias	Low risk of bias	Low risk of bias	Low risk of bias
Raja 2003	Low risk of bias	Low risk of bias	Low risk of bias	Low risk of bias	Low risk of bias
Muneretto 2003	Unclear risk of bias	Unclear risk of bias	Low risk of bias	Low risk of bias	Low risk of bias
Lingaas 2004/Lingass 2006	Unclear risk of bias	Low risk of bias	Low risk of bias	Low risk of bias	Low risk of bias
Legare 2004/Karolak 2007	Low risk of bias	Low risk of bias	Low risk of bias	Low risk of bias	Low risk of bias
PRAGUE-4	Unclear risk of bias	Unclear risk of bias	Low risk of bias	Low risk of bias	Low risk of bias
Gerola 2004	Unclear risk of bias	Unclear risk of bias	Low risk of bias	Low risk of bias	Low risk of bias
Khan 2004	Unclear risk of bias	Unclear risk of bias	Low risk of bias	Low risk of bias	Low risk of bias
Chen 2004	High risk of bias	High risk of bias	Low risk of bias	Low risk of bias	Low risk of bias
Octopus	Low risk of bias	Low risk of bias	Low risk of bias	Low risk of bias	Low risk of bias
SMART	Low risk of bias	Low risk of bias	Low risk of bias	Low risk of bias	Low risk of bias
JOCRI	Low risk of bias	Low risk of bias	Low risk of bias	Low risk of bias	Low risk of bias
Motallebazadeh 2007	Low risk of bias	Low risk of bias	Low risk of bias	Low risk of bias	Low risk of bias
Hernandez 2007	Low risk of bias	Low risk of bias	Low risk of bias	Low risk of bias	Low risk of bias
Nogueira 2008	Unclear risk of bias	Unclear risk of bias	Low risk of bias	Low risk of bias	High risk of bias
Naseri 2009	Unclear risk of bias	Unclear risk of bias	Low risk of bias	Low risk of bias	Low risk of bias
ROOBY	Low risk of bias	Low risk of bias	Low risk of bias	Low risk of bias	Low risk of bias
Iqbal 2014	Unclear risk of bias	Unclear risk of bias	Low risk of bias	Low risk of bias	Low risk of bias
PROMISS	Low risk of bias	Low risk of bias	Low risk of bias	Low risk of bias	Low risk of bias
MASS III	Low risk of bias	Low risk of bias	Low risk of bias	Low risk of bias	Low risk of bias
BBS	Low risk of bias	Low risk of bias	Low risk of bias	Low risk of bias	Low risk of bias
on-off	Low risk of bias	Low risk of bias	Low risk of bias	Low risk of bias	Low risk of bias
DOORS	Low risk of bias	Low risk of bias	Low risk of bias	Low risk of bias	Low risk of bias
CORONARY	Low risk of bias	Low risk of bias	Low risk of bias	Low risk of bias	Low risk of bias

Lei 2014	Unclear risk of bias	Low risk of bias	Low risk of bias	Low risk of bias	Low risk of bias
PRAGUE-6	Unclear risk of bias	Low risk of bias	Low risk of bias	Low risk of bias	Low risk of bias
GOPCABE	Low risk of bias	Low risk of bias	Low risk of bias	Low risk of bias	Low risk of bias

For performance bias and detection bias (i.e., blinding of participants and personnel, and blinding of outcome assessment), we judged all randomized controlled trials as having a lower risk of bias. We made this judgement based on the following rational: Given the nature of comparing on-pump CABG with off-pump CABG, achieving a double-blinded trial was challenging. Additionally, all endpoints evaluated in these trials were objective in nature, further reducing the potential for bias.

Table S10. The outcomes of Begg's test

	Outcome	Studies	Begg's test
Short-term	Mortality	25	0.6073
	Stroke	23	0.6532
	Coronary reintervention	7	NA
	Acute renal failure requiring dialysis	11	1.0000
Mid-term	Mortality	11	0.4363
	Stroke	7	NA
	Coronary reintervention	9	NA
Long-term	Mortality	8	NA
	Stroke	3	NA
	Coronary reintervention	6	NA

NA: Not applicable

Table S11. Outcome definition

	Mortality	Myocardial infarction	Stroke	Coronary reintervention	Acute renal failure requiring dialysis
Short-term outcome					
Van Dijk 2001	√	MI-definition-1	Stroke-definiton-1	√	×
Angelini 2002	√	MI-definition-2	×	×	×
Muneretto 2003	√	No definition	No definition	×	×
Raja 2003	√	×	×	×	×
Puskas 2003	√	No definition	No definition	√	√
Chen 2004	√	No definition	No definition	×	×
Lingaas 2004	√	×	No definition	×	×
Legare 2004	√	MI-definition-3	No definition	×	×
Straka 2004	√	MI-definition-4	Stroke-definiton-2	×	√
Gerola 2004	√	MI-definition-2	No definition	×	×
Khan 2004	√	No-definition	×	×	×
Kobayashi 2005	√	MI-definition-5	No definition	√	√
Motallebzadeh 2007	√	×	No definition	×	×
Hernandez 2007	√	×	Stroke-definition-2	×	×
Naseri 2009	×	×	No-definition	×	×
Shroyer 2009	√	×	No-definition	×	√
Sousa Uva 2010	√	No- definition	No- definition	×	√
Hueb 2010	√	MI-definition-6	Stroke-definition-1	×	√
Moller 2010	√	MI-definition-7	Stroke-definition-3	√	√
Lemma 2012	√	MI-definition-8	Stroke-definition-3	×	×
Houlind 2012	√	MI-definition-9	Stroke-definition-3	×	×
Lamy 2012	√	MI-definiton-10	Stroke-definiton-3	√	√
Diegeler 2013	√	MI-definition-11	Stroke-definition-3	√	√
Iqbal 2014	√	No definition	Stroke-definition-3	×	×
Lei 2014	√	No definition	No definition	×	√
Hlavicka 2016	√	MI-definition-12	Stroke-definition-1	√	√
Mid-term outcome					
Angelini 2002	√	MI-definition-2	No definition	√	NA
Muneretto 2003	√	No definition	×	×	NA
Nathoe 2003	√	MI-definition-1	Stroke-definiton-1	√	NA
Puskas 2004	√	No definition	No definition	√	√
Lingaas 2006	√	×	×	√	NA
Nogueira 2008	√	No definition	No definition	×	NA
Shroyer 2009	√	No definition	×	√	NA
Sousa Uva 2010	√	×	×	√	NA
Lamy 2013	√	MI-definiton-10	Stroke-definiton-3	√	NA
Diegeler 2013	√	MI-definition-11	Stroke-definition-3	√	NA
Hlavicka 2016	√	MI-definition-12	Stroke-definition-3	√	NA
Long-term outcome					
Angelini 2002	√	×	×	×	NA
Karolak 2007	√	×	×	√	NA
Van Dijk 2007	√	MI-definition-1	Stroke-definiton-1	√	NA
Hueb 2010	√	MI-definition-6	Stroke-definition-1	√	NA
Puskas 2011	√	×	×	×	NA

Lamy 2016	√	MI-definiton-10	Stroke-definiton-3	√	NA
Shroyer 2017	√	MI-definition-13	×	√	NA
Diegeler 2019	√	No definition	×	√	NA
Quin 2022	√	×	×	√	NA

MI denotes myocardial infarction; NA denotes not applicable
Clinical diagnostic criteria for Myocardial infarction (MI)

MI-definiton-1:
Perioperative MI (within 7 days of surgery) was defined as CK-MB to >5*99th percent URL. Non-perioperative MI (later than 7 days of surgery), it was considered present when 2 of the following criteria were met: 1. chest discomfort lasting >30 minutes; 2.CK-MB/CK ratio >0.1; 3.the development of abnormal new Q waves on the ECG.

MI-definition-2:
New Q waves of >0.04ms and/or a reduction in R waves >25% in ≥2 leads.

MI-definition-3:
New Q waves of >0.04ms in ≥2 leads or ECG ST changes in association with significant CK-MB enzyme release.

MI-definition-4:
ECG sign of necrosis, CK and CK-MB elevation, or new akinetic segment at echocardiogram.

MI-definition-5:
CK-MB measurement ≥100 IU/L with ECG change (new Q wave or T inversion).

MI-definition-6:
Perioperative MI (within 7 days of surgery) was defined as CK-MB measurement ≥5*99th percent URL (>30 ng/mL) or troponin I values >5 ng/mL whether new Q waves is present.

MI-definition-7:
Perioperative MI (within 48 hours of surgery) was defined as CK–MB increase >80 g/L or troponin T >3.0 g/L. Non-perioperative MI (later than 48 hours of surgery) was defined as CK–MB >10 g/L or troponin T >0.1 g/L together with at least 1 of the following findings: 1. symptoms of ischemia; or 2. ECG changes indicative of new ischemia (new ST-T changes or new LBBB); or 3. development of pathological Q waves in the ECG; 4.coronary reintervention.

MI-definition-8:
The appearance of a new Q wave on the ECG with CK-MB measurement ≥100 UI/L and/or CK-MB/CK ratio >0.1 and/or with documented new wall motion abnormalities other than septal on the echocardiogram.

MI-definition-9:
Perioperative MI (within 72 hours of surgery) was defined as CK-MB measurement ≥75 ng/ml or new Q-wave evidence of MI, along with CK-MB of ≥50 ng/ml. Non-perioperative MI (later than 72 hours after surgery) was defined as new Q-wave evidence of MI by day 30 that was not present at day 3, or MI as defined by the World Health Organization.

MI-definiton-10:
Perioperative MI (within 72 hours of surgery) was defined as CK-MB measurement ≥5*99th percent URL whether new Q waves or new LBBB is present. Non-perioperative MI (later than 72 hours after surgery) was defined as a detection of rise and/or fall of cardiac biomarkers with at least 1 value above the 99th percentile of the URL together with evidence of myocardial ischemia with at least 1 of the following: 1. symptoms of ischemia; or 2. ECG changes indicative of new ischemia (new ST-T changes or new LBBB); or 3. development of pathological Q waves in the ECG.

MI-definition-11:
Perioperative MI (within 72 hours of surgery) was defined as the observation of at least one of the following three criteria: 1. CK-MB measurement ≥ 5*99th percentile URL; or 2. angiographic evidence of new graft or native coronary artery occlusion; or 3. imaging evidence of new loss of viable myocardium. Non-perioperative MI (later than 72 hours after surgery) was defined as a rise of cardiac biomarkers with at least one value above the 99th percentile of the URL in conjunction with evidence of myocardial ischemia with at least one of the following: 1. symptoms of ischemia; or 2. ECG changes indicative of new ischemia (new ST-T changes or new LBBB); or 3. development of pathological Q waves in the ECG; or 4. imaging evidence of new loss of viable myocardium or new regional wall motion abnormality.

MI-definition-12:
Perioperative MI (within 72 hours of surgery) was as the observation of at least one of the following four criteria:1. CK-MB or CK-MB mass measurement ≥ 5*99th percentile URL; 2. new pathological Q-waves in two or more contiguous ECG leads, or a new LBBB; 3. angiographic evidence of new graft or native coronary artery occlusion; 4. imaging evidence of new loss of viable myocardium. Non-perioperative MI (later than 72 hours after surgery) was defined as a rise of cardiac biomarkers with at least one value above the 99th percentile of the URL in conjunction with evidence of myocardial ischemia with at least one of the following: 1. symptoms of ischemia; or 2. ECG changes indicative of new ischemia (new ST-T changes or new LBBB); or 3. development of pathological Q waves in the ECG; or 4. imaging evidence of new loss of viable myocardium or new regional wall motion abnormality.

MI-definition-13:

A detection of rise and/or fall of cardiac biomarkers with at least 1 value above the 99th percentile of the URL together with evidence of myocardial ischemia with at least 1 of the following: 1. symptoms of ischemia; or 2. ECG changes indicative of new ischemia (new ST-T changes or new LBBB); or 3. development of pathological Q waves in the ECG; or 4. imaging evidence of new loss of viable myocardium or new regional wall motion abnormality.

Clinical diagnostic criteria for stroke

Stroke-definition-1 was focal brain injury persisting for >24 hours, combined with an increase in handicap of at least 1 grade on the Rankin scale

Stroke-definition-2 was global or focal neurologic deficit persisting for >24 hours

Stroke-definition-3 was global or focal neurologic deficit persisting for >24 hours, and verified by a neurologist and/or an imagine method (CT or MRI)

Coronary reintervention included redo coronary artery graft bypass (redo CABG) or percutaneous coronary intervention (PCI)

Table S12. Prior meta-analysis which only included randomized controlled trials

Author/Year	Studies	Participants	Endpoint	Conclusions:
Parolari 2003	9 RCTs	1090	Short-term major outcomes (death, stroke, or myocardial infarction)	A trend toward reduction in the risk of short-term major outcomes for patients in OPCABG.
Parolari 2005	5 RCTs	1105	Overall graft patency	A reduction in postoperative graft patency in the OPCABG groups of patients.
Takagi 2007	8 RCTs	811	Cognitive outcomes	Compared with ONCABG, there were better cognitive outcomes 1 months to 3 months but these benefits lacked within 2 weeks and 6 months to 12 months in OPCABG
Takagi 2007	32 RCTs	3714	Short-term stroke	OPCABG did not reduce stroke, compared with results of ONCABG.
Marasco 2008	8 RCTs	892	Cognitive outcomes	There were no convincing differences in outcomes in neurocognitive testing between OPCABG and ONCABG.
Moller 2008	66 RCTs	5537	Overall mortality, stroke and coronary reintervention	There were no statistically significant differences regarding mortality, stroke and coronary reintervention between OPCABG and ONCABG.
Takagi 2008	9 RCTs	1012	Cognitive outcomes	Compared with ONCABG, there were better cognitive outcomes 1 months to 3 months but these benefits lacked within 2 weeks and 6 months to 12 months in OPCABG.
Feng 2009	10 RCTs	1018	Mid-term mortality, stroke and coronary reintervention	Compared with ONCABG, OPCABG did not significantly reduce mid-term mortality, stroke and coronary reintervention.
Seabra 2010	22 RCTs	4819	AKI, dialysis requirement	Off-pump CABG may be associated with a lower incidence of postoperative AKI but may not affect dialysis requirement
Takagi 2010	12 RCTs	4326	Late (> or = 1 year) all-cause mortality	Compared to ONCABG, OPCABG may increase late all-cause mortality.
Takagi 2010	8 RCTs	3702	Graft patency (> or = 3 months)	Compared to ONCABG, OPCABG may increase overall graft occlusion, especially saphenous vein occlusion.
Afilalo 2012	59 RCTs	8961	Short-term mortality, stroke	There appeared to be a beneficial effect of OPCABG on short-term stroke, while there was no significant difference in short-term mortality.
Chen 2012	43 RCTs	16828	Short-term stroke	There was no significant difference in the incidences of stroke between OPCABG and ONCABG.
Sa 2012	47 RCTs	13524	Short-term mortality, stroke	Compared to ONCABG, OPCABG reduced the incidence of short-term stroke but had no substantial effect on mortality.
Sun 2012	13 RCTs	2326	Postoperative cognitive dysfunction	Compared to ONCABG, OPCAB was found to be associated with a reduced incidence of early-stage postoperative cognitive dysfunction
Takagi 2013	12 RCTs	11594	Coronary reintervention (> or = 1 year)	Compared to ONCABG, OPCABG may increase repeat revascularization rates.
Zhang 2013	9 RCTs	10459	Mid-term mortality	Compared to ONCABG, OPCABG did not increase mid-term mortality.
Chaudhry 2014	3 RCTs	879	Mid-term mortality (>1 year and < or = 5 years)<br>Long-term mortality (> 5 years)	There was comparable mid-term and long-term mortality between OPCABG and ONCABG.
Takagi 2014	5 RCTs	1486	Long-term mortality (> 5 years)	Compared to ONCABG, there was a trend towards an increased long-term mortality in OPCABG.
Takagi 2014	8 RCTs	10954	Mid-term MACCE (> or = 1 year)	OPCABG appeared not to increase mid-term MACCE over ONCABG.
Zhang 2014	12 RCTS	4711	Overall graft Patency	Compared to OPCABG, ONCABG reduced the incidence of SVG graft occlusion significantly but did not affect left internal mammary artery and radial

				artery graft patency.
Luo 2015	7 RCTS	9128	Short-term mortality, stroke, renal failure and coronary reintervention	There was no difference in the short-term outcomes between ONCABG and OPCABG.
Deppe 2016	51 RCTs	16904	Short-term mortality, stroke, coronary reintervention and renal dysfunction	There was no difference regard short-term mortality between ONCABG and OPCABG. While the need for coronary reintervention was increased after OPCABG, ONCABG was associated with an increased occurrence of stroke, renal impairment.
Dieberg 2016	54 RCTs	16261	Short-term mortality, stroke	There was no difference regard short-term mortality between OPCABG and ONCABG, while there was a trend towards a reduced incidence of stroke in OPCABG.
Kowalewski 2016	100 RCTs	19192	Short-term mortality, stroke	There was no difference regard short-term mortality, while OPCABG was associated with a significant reduction in the odds of OPCABG in terms of short-term mortality.
Filardo 2018	36 RCTs	13338	Short-term mortality<br>Late mortality (> or = 1 year)	Evidence from RCTs showed no differences regarding short-term and late mortality between OPCABG and ONCABG.
Gaudino 2018	104 RCTs	20626	All-cause mortality at the longest reported follow-up	OPCABG was associated with decreased midterm survival compared with ONCABG
Smart 2018	6 RCTs	8145	Long-term mortality, stroke and coronary reintervention	There was no difference regard long-term stroke and coronary reintervention, while OPCABG increased the long-term mortality compared with ONCABG.
Takagi 2018	8 RCTs	8780	Long-term mortality	Compared to ONCABG, OPCABG increased long-term mortality.
Zhou 2018	11 RCTs	11246	Short, mid and long-term coronary reintervention	Compared with ONCABG, OPCABG increases mid-term coronary reintervention rate, but does not affect long-term coronary reintervention rate.
Mauldon 2020	37 RCTs	15324	Short-term mortality, stroke	There was a significant odds reduction for patients receiving OPCABG suffering a stroke, while there were no significant differences in the odds of short-term mortality.
Machado 2022	5 RCTs	6221	Short-term and late (the longest follow-up) mortality, stroke, coronary reintervention and renal complication	Compared to ONCABG, OPCABG was associated with a higher risk of short-term coronary reintervention, while there was no difference in other short-term or late outcomes.
Zhou 2022	16 RCTs	6227	Overall graft patency	Compared to ONCABG, graft patency was decreased in OPCABG for overall grafts, SVG grafts.

AKI denotes acute kidney injury. RCTs denotes randomized controlled trials. OPCABG denotes off-pump coronary bypass grafting. ONCABG denotes on-pump coronary bypass grafting. MACCE denotes Major cardio- and cerebrovascular events. SVG denotes saphenous vein graft.

Figure S1. Flow of diagram

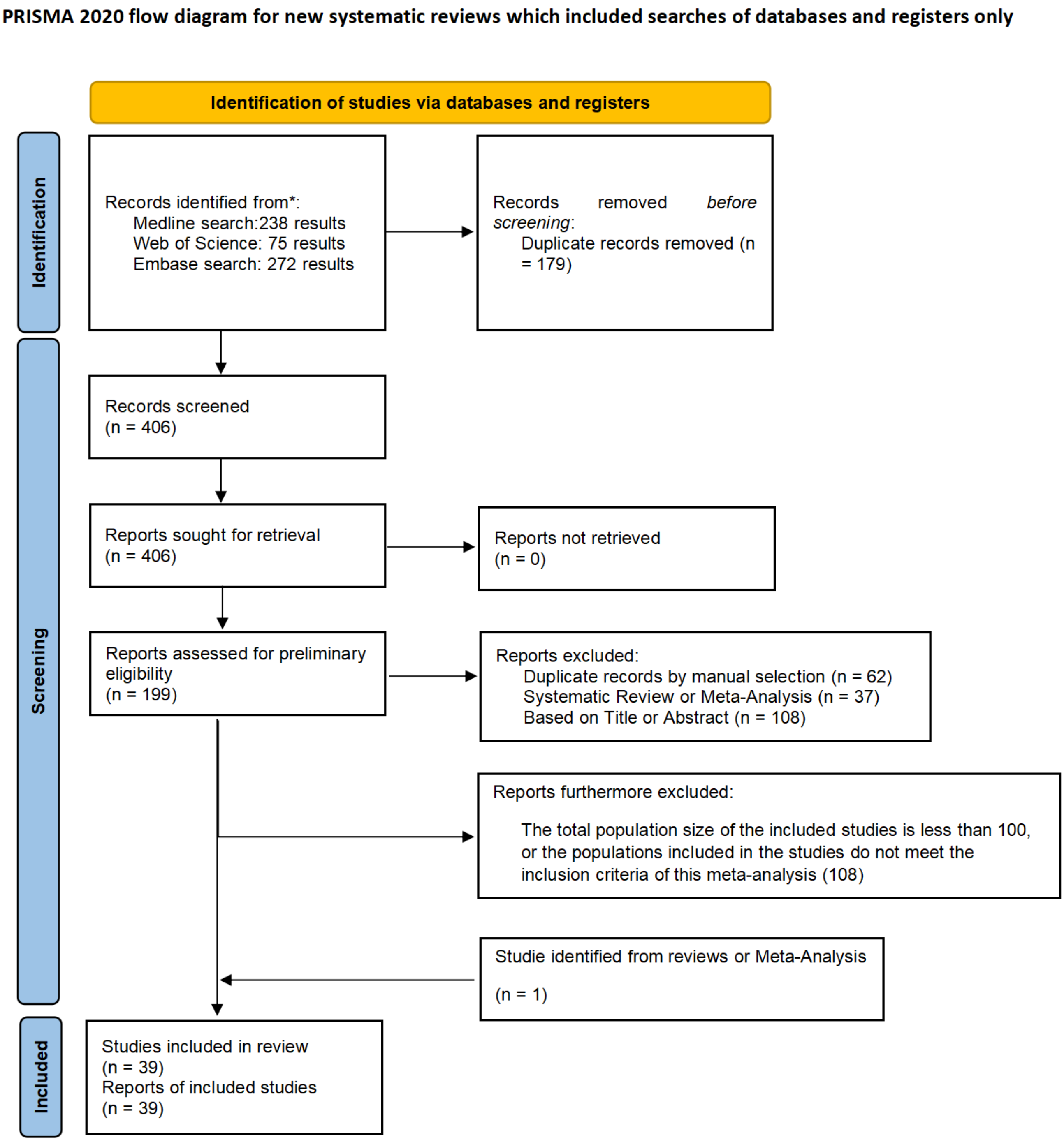

*Consider, if feasible to do so, reporting the number of records identified from each database or register searched (rather than the total number across all databases/registers).

**If automation tools were used, indicate how many records were excluded by a human and how many were excluded by automation tools.

Figure S2. Forest plot of number of anastomoses/grafts per patient

Figure S2.1 No of anastomoses/grafts per patient (Fixed effect)

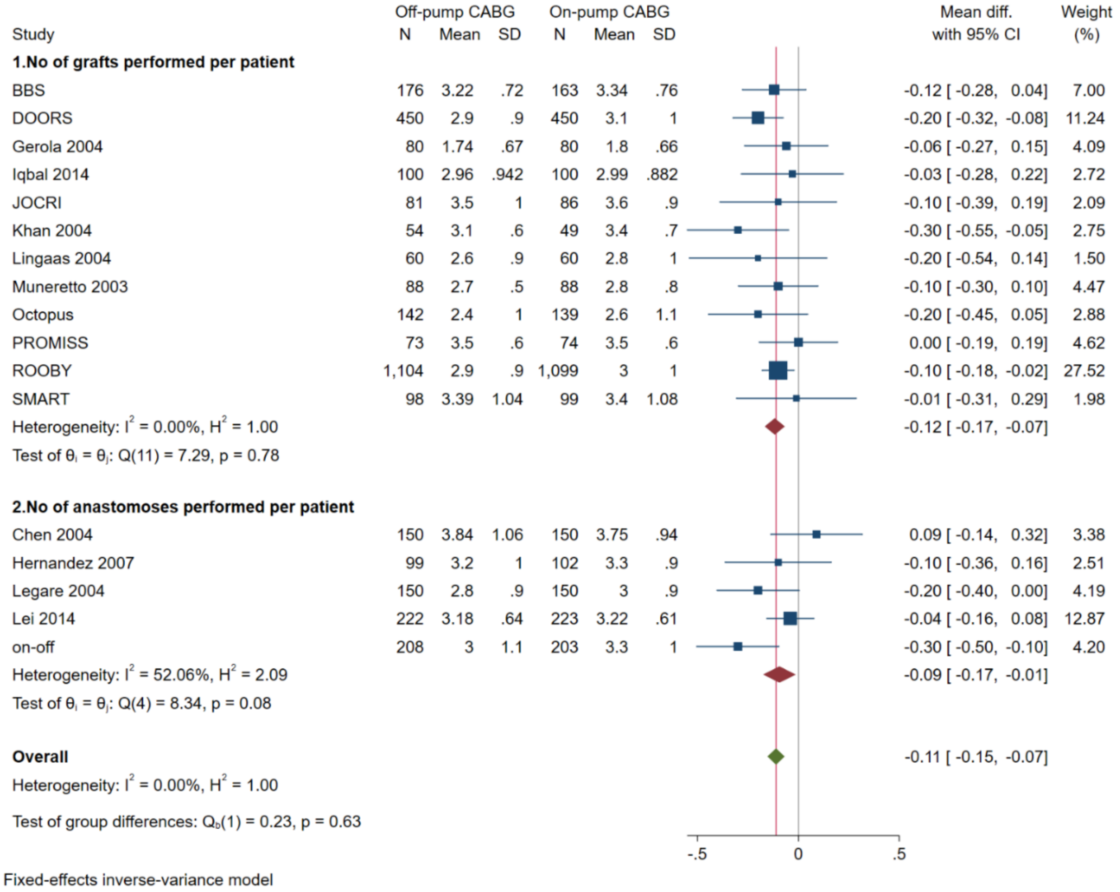

Figure S2.2 No of anastomoses/grafts per patient (Random effect)

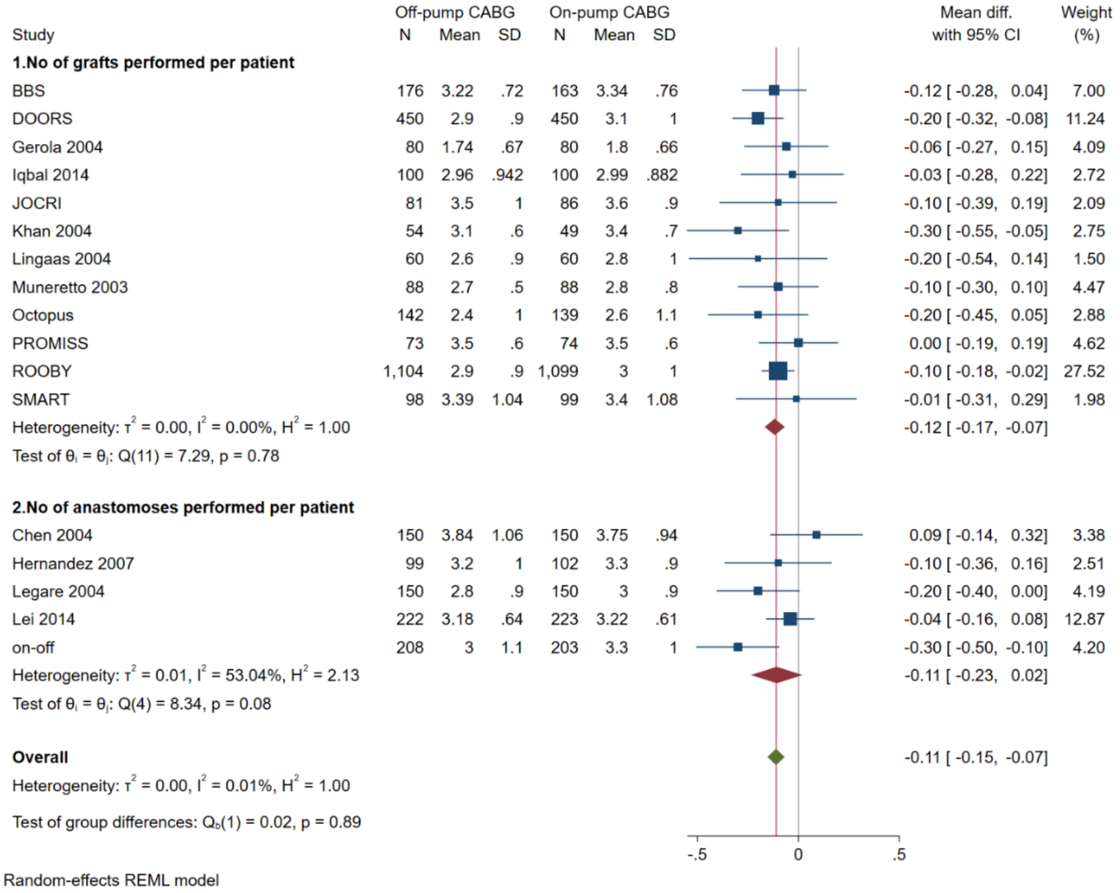

Figure S3. Forest plot of clinical outcome (Fixed effect)

Figure S3.1 Short-term Stroke

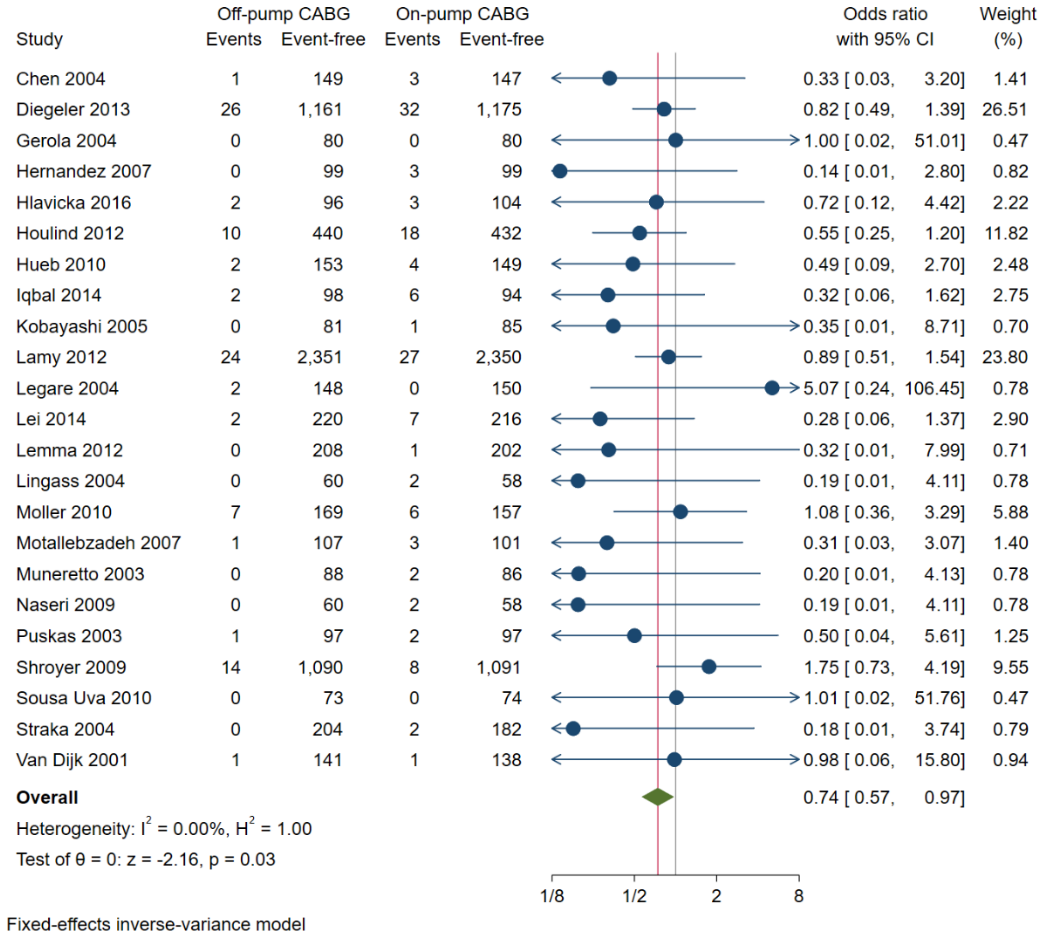

Figure S3.2 Long-term Mortality

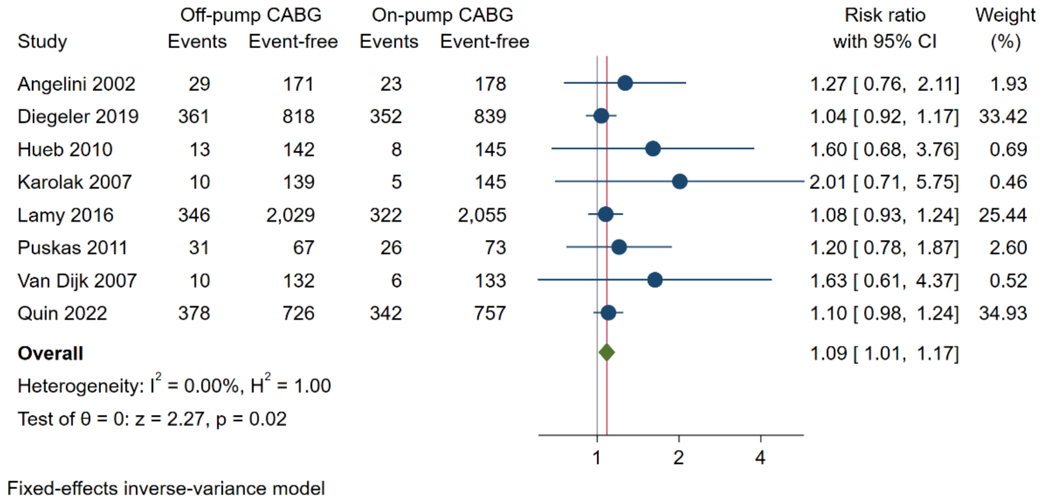

Figure S3.3 Mid-term Stroke

Study	Off-pump CABG Events	Off-pump CABG Event-free	On-pump CABG Events	On-pump CABG Event-free	Risk ratio with 95% CI	Weight (%)
Angelini 2002	3	197	6	195	0.50 [0.13, 1.98]	4.19
Diegeler 2013	41	1,138	52	1,139	0.80 [0.53, 1.19]	48.92
Hlavicka 2016	2	96	5	102	0.44 [0.09, 2.20]	3.01
Lamy 2013	36	2,339	40	2,337	0.90 [0.58, 1.41]	39.50
Nathoe 2003	1	141	2	137	0.49 [0.04, 5.34]	1.38
Nogueira 2008	0	105	3	94	0.13 [0.01, 2.52]	0.91
Puskas 2004	2	88	2	93	1.06 [0.15, 7.34]	2.10
**Overall**					0.79 [0.60, 1.05]	

Heterogeneity: I^2 = 0.00%, H^2 = 1.00

Test of θ = 0: z = -1.63, p = 0.10

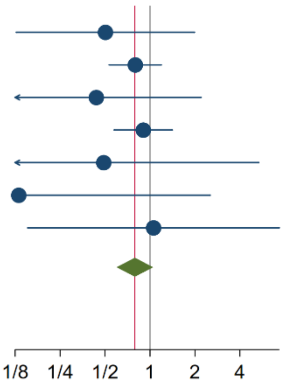

Fixed-effects inverse-variance model

Figure S3.4 Long-term Stroke

Study	Off-pump CABG Events	Off-pump CABG Event-free	On-pump CABG Events	On-pump CABG Event-free	Risk ratio with 95% CI	Weight (%)
Hueb 2010	3	152	5	148	0.59 [0.14, 2.44]	5.63
Lamy 2016	55	2,320	66	2,311	0.83 [0.59, 1.19]	90.10
Van Dijk 2007	2	140	5	134	0.39 [0.08, 1.98]	4.27
**Overall**					0.79 [0.57, 1.11]	

Heterogeneity: I^2 = 0.00%, H^2 = 1.00

Test of θ = 0: z = -1.36, p = 0.17

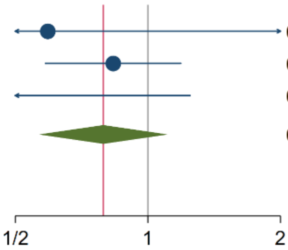

Fixed-effects inverse-variance model

Figure S3.5 Short-term Mortality

Study	Off-pump CABG Events	Off-pump CABG Event-free	On-pump CABG Events	On-pump CABG Event-free	Odds ratio with 95% CI	Weight (%)
Angelini 2002	0	200	2	199	0.20 (0.01, 4.17)	0.55
Chen 2004	1	149	2	148	0.50 (0.04, 5.54)	0.87
Diegeler 2013	31	1,156	34	1,173	0.93 (0.56, 1.52)	20.75
Gerola 2004	1	79	3	77	0.32 (0.03, 3.19)	0.97
Hernandez 2007	0	99	1	101	0.34 (0.01, 8.45)	0.49
Hlavicka 2016	4	94	6	101	0.72 (0.20, 2.62)	3.01
Houlind 2012	7	443	8	442	0.87 (0.31, 2.43)	4.83
Hueb 2010	1	154	0	153	2.98 (0.12, 73.74)	0.49
Iqbal 2014	2	98	3	97	0.66 (0.11, 4.04)	1.54
Khan 2004	0	54	0	49	0.91 (0.02, 46.64)	0.33
Kobayashi 2005	0	81	0	86	1.06 (0.02, 54.12)	0.33
Lamy 2012	60	2,315	59	2,318	1.02 (0.71, 1.47)	38.14
Legare 2004	2	148	1	149	2.01 (0.18, 22.45)	0.87
Lei 2014	2	220	7	216	0.28 (0.06, 1.37)	2.02
Lemma 2012	4	204	7	196	0.55 (0.16, 1.90)	3.26
Lingass 2004	1	59	1	59	1.00 (0.06, 16.37)	0.65
Moller 2010	6	170	11	152	0.49 (0.18, 1.35)	4.87
Motallebzadeh 2007	1	107	2	102	0.48 (0.04, 5.34)	0.87
Muneretto 2003	3	85	2	86	1.52 (0.25, 9.31)	1.53
Puskas 2003	1	97	2	97	0.50 (0.04, 5.61)	0.86
Raja 2003	0	150	9	141	0.05 (0.00, 0.86)	0.62
Shroyer 2009	18	1,086	13	1,086	1.38 (0.68, 2.84)	9.79
Sousa Uva 2010	0	73	0	74	1.01 (0.02, 51.76)	0.33
Straka 2004	4	200	2	182	1.82 (0.33, 10.05)	1.73
Van Dijk 2001	0	142	0	139	0.98 (0.02, 49.68)	0.33
**Overall**					0.89 (0.71, 1.12)	

Heterogeneity: $I^2 = 0.00\%$, $H^2 = 1.00$

Test of θ = 0: z = -1.00, p = 0.32

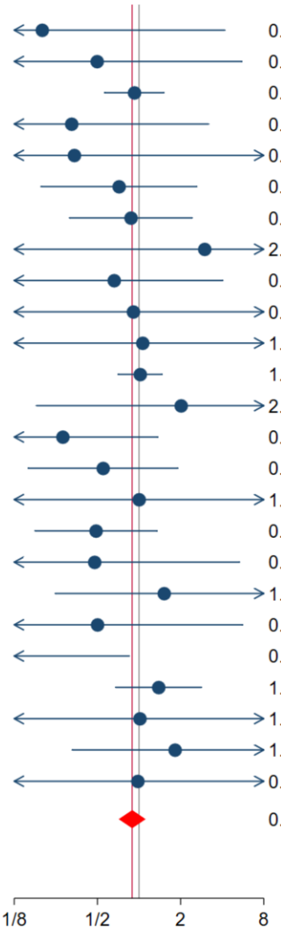

Fixed-effects inverse-variance model

Figure S3.6 Mid-term Mortality

Study	Off-pump CABG Events	Off-pump CABG Event-free	On-pump CABG Events	On-pump CABG Event-free	Risk ratio with 95% CI	Weight (%)
Angelini 2002	4	196	7	194	0.57 [0.17, 1.93]	1.78
Diegeler 2013	83	1,096	95	1,096	0.88 [0.66, 1.17]	32.70
Hlavicka 2016	16	82	11	96	1.59 [0.78, 3.25]	5.10
Lamy 2013	122	2,253	119	2,258	1.03 [0.80, 1.31]	43.34
Lingaas 2006	1	59	1	59	1.00 [0.06, 15.62]	0.35
Muneretto 2003	4	84	4	84	1.00 [0.26, 3.87]	1.43
Nathoe 2003	2	140	2	137	0.98 [0.14, 6.85]	0.69
Nogueira 2008	0	105	0	97	0.92 [0.02, 46.15]	0.17
Puskas 2004	4	86	4	91	1.06 [0.27, 4.09]	1.43
Shroyer 2009	43	1,018	30	1,020	1.42 [0.90, 2.24]	12.49
Sousa Uva 2010	1	72	3	71	0.34 [0.04, 3.17]	0.52
**Overall**					1.02 [0.87, 1.20]	

Heterogeneity: $I^2 = 0.00\%$, $H^2 = 1.00$

Test of θ = 0: z = 0.27, p = 0.79

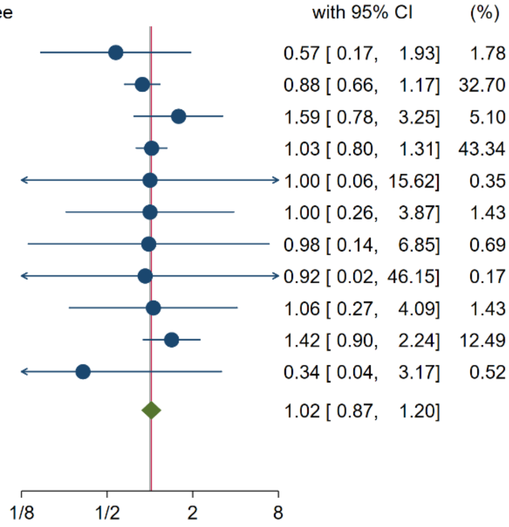

Fixed-effects inverse-variance model

Figure S3.7 Short-term Coronary reintervention

Study	Off-pump CABG Events	Off-pump CABG Event-free	On-pump CABG Events	On-pump CABG Event-free	Odds ratio with 95% CI	Weight (%)
Diegeler 2013	15	1,172	5	1,202	3.08 [1.11, 8.49]	40.54
Hlavicka 2016	1	97	1	106	1.09 [0.07, 17.71]	5.39
Kobayashi 2005	0	81	0	86	1.06 [0.02, 54.12]	2.70
Lamy 2012	16	2,359	4	2,373	4.02 [1.34, 12.05]	34.72
Moller 2010	1	175	3	160	0.30 [0.03, 2.96]	8.09
Puskas 2003	0	98	1	98	0.33 [0.01, 8.28]	4.05
Van Dijk 2001	2	140	0	139	4.96 [0.24, 104.34]	4.51
**Overall**					2.40 [1.26, 4.59]	

Heterogeneity: I^2 = 6.09%, H^2 = 1.06

Test of θ = 0: z = 2.66, p = 0.01

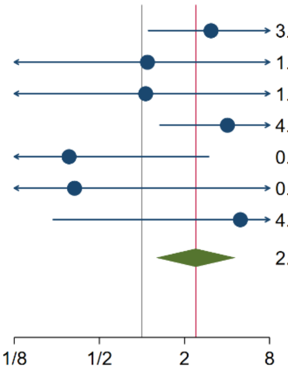

Fixed-effects inverse-variance model

Figure S3.8 Mid-term Coronary reintervention

Study	Off-pump CABG Events	Off-pump CABG Event-free	On-pump CABG Events	On-pump CABG Event-free	Risk ratio with 95% CI	Weight (%)
Angelini 2002	4	196	4	197	1.00 [0.25, 3.96]	3.40
Diegeler 2013	36	1,143	24	1,167	1.52 [0.91, 2.52]	24.57
Hlavicka 2016	5	93	3	104	1.82 [0.45, 7.42]	3.24
Lamy 2013	33	2,342	20	2,357	1.65 [0.95, 2.87]	20.95
Lingaas 2006	7	53	3	57	2.33 [0.63, 8.60]	3.76
Nathoe 2003	7	135	4	135	1.71 [0.51, 5.72]	4.40
Puskas 2004	2	88	1	94	2.11 [0.19, 22.88]	1.13
Shroyer 2009	49	1,012	36	1,014	1.35 [0.88, 2.05]	35.96
Sousa Uva 2010	3	70	3	71	1.01 [0.21, 4.86]	2.60
**Overall**					1.49 [1.16, 1.92]	

Heterogeneity: I^2 = 0.00%, H^2 = 1.00

Test of θ = 0: z = 3.09, p = 0.00

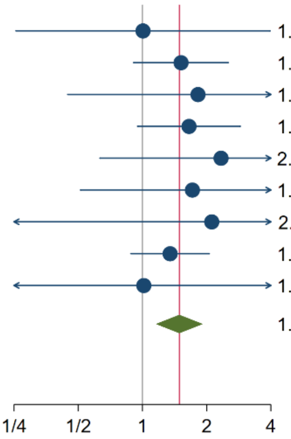

Fixed-effects inverse-variance model

Figure S3.9 Long-term Coronary reintervention

Study	Off-pump CABG Yes	Off-pump CABG No	On-pump CABG Yes	On-pump CABG No	Risk ratio with 95% CI	Weight (%)
Diegeler 2019	43	1,025	34	1,023	1.25 [0.80, 1.95]	10.30
Hueb 2010	10	155	9	153	1.09 [0.46, 2.61]	2.63
Karolak 2007	2	149	4	150	0.51 [0.09, 2.74]	0.71
Lamy 2016	66	2,375	55	2,377	1.20 [0.84, 1.70]	16.09
Quin 2022	227	1,104	210	1,099	1.06 [0.90, 1.26]	68.38
Van Dijk 2007	11	142	5	139	2.07 [0.74, 5.81]	1.89
**Overall**					1.11 [0.96, 1.28]	

Heterogeneity: I^2 = 0.00%, H^2 = 1.00

Test of θ = 0: z = 1.45, p = 0.15

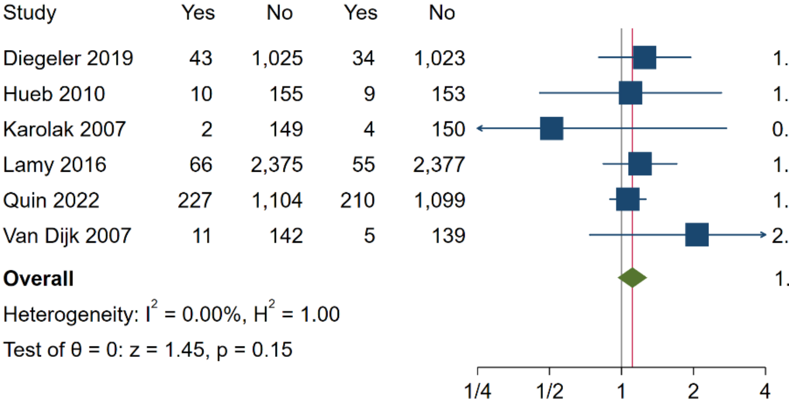

Fixed-effects inverse-variance model

Figure S3.10 Short-term Acute renal failure requiring dialysis

Study	Off-pump CABG Events	Off-pump CABG Event-free	On-pump CABG Events	On-pump CABG Event-free	Odds ratio with 95% CI	Weight (%)
Diegeler 2013	29	1,158	37	1,170	0.79 [0.48, 1.30]	37.29
Hlavicka 2016	1	97	5	102	0.21 [0.02, 1.83]	1.93
Hueb 2010	1	154	1	152	0.99 [0.06, 15.92]	1.17
Kobayashi 2005	0	81	1	85	0.35 [0.01, 8.71]	0.88
Lamy 2012	28	2,347	27	2,350	1.04 [0.61, 1.77]	32.03
Lei 2014	2	220	5	218	0.40 [0.08, 2.06]	3.32
Moller 2010	7	169	8	155	0.80 [0.28, 2.26]	8.41
Puskas 2003	2	96	0	99	5.16 [0.24, 108.78]	0.97
Shroyer 2009	9	1,095	10	1,089	0.90 [0.36, 2.21]	11.07
Sousa Uva 2010	0	73	0	74	1.01 [0.02, 51.76]	0.59
Straka 2004	2	202	2	182	0.90 [0.13, 6.46]	2.33
**Overall**					0.85 [0.63, 1.15]	

Heterogeneity: $I^2 = 0.00\%$, $H^2 = 1.00$

Test of $\theta = 0$: $z = -1.06$, $p = 0.29$

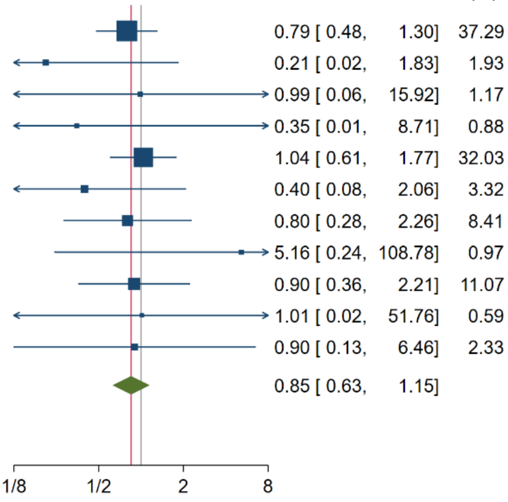

Fixed-effects inverse-variance model

Figure S4. Forest plot of clinical outcome (Random effect)

Figure S4.1 Short-term Stroke

Study	Off-pump CABG Events	Off-pump CABG Event-free	On-pump CABG Events	On-pump CABG Event-free	Odds ratio with 95% CI	Weight (%)
Chen 2004	1	149	3	147	0.33 (0.03, 3.20)	1.41
Diegeler 2013	26	1,161	32	1,175	0.82 (0.49, 1.39)	26.51
Gerola 2004	0	80	0	80	1.00 (0.02, 51.01)	0.47
Hernandez 2007	0	99	3	99	0.14 (0.01, 2.80)	0.82
Hlavicka 2016	2	96	3	104	0.72 (0.12, 4.42)	2.22
Houlind 2012	10	440	18	432	0.55 (0.25, 1.20)	11.82
Hueb 2010	2	153	4	149	0.49 (0.09, 2.70)	2.48
Iqbal 2014	2	98	6	94	0.32 (0.06, 1.62)	2.75
Kobayashi 2005	0	81	1	85	0.35 (0.01, 8.71)	0.70
Lamy 2012	24	2,351	27	2,350	0.89 (0.51, 1.54)	23.80
Legare 2004	2	148	0	150	5.07 (0.24, 106.45)	0.78
Lei 2014	2	220	7	216	0.28 (0.06, 1.37)	2.90
Lemma 2012	0	208	1	202	0.32 (0.01, 7.99)	0.71
Lingass 2004	0	60	2	58	0.19 (0.01, 4.11)	0.78
Moller 2010	7	169	6	157	1.08 (0.36, 3.29)	5.88
Motallebzadeh 2007	1	107	3	101	0.31 (0.03, 3.07)	1.40
Muneretto 2003	0	88	2	86	0.20 (0.01, 4.13)	0.78
Naseri 2009	0	60	2	58	0.19 (0.01, 4.11)	0.78
Puskas 2003	1	97	2	97	0.50 (0.04, 5.61)	1.25
Shroyer 2009	14	1,090	8	1,091	1.75 (0.73, 4.19)	9.55
Sousa Uva 2010	0	73	0	74	1.01 (0.02, 51.76)	0.47
Straka 2004	0	204	2	182	0.18 (0.01, 3.74)	0.79
Van Dijk 2001	1	141	1	138	0.98 (0.06, 15.80)	0.94
**Overall**					0.74 (0.57, 0.97)	

Heterogeneity: $\tau^2 = 0.00$, $I^2 = 0.00\%$, $H^2 = 1.00$

Test of $\theta = 0$: $z = -2.16$, $p = 0.03$

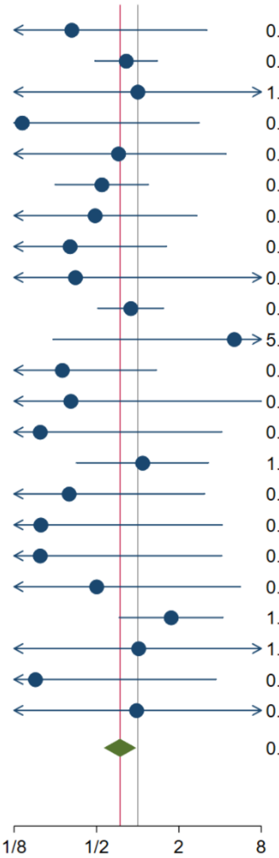

Random-effects REML model

Figure S4.2 Long-term Mortality

Study	Off-pump CABG Events	Off-pump CABG Event-free	On-pump CABG Events	On-pump CABG Event-free	Risk ratio with 95% CI	Weight (%)
Angelini 2002	29	171	23	178	1.27 [0.76, 2.11]	1.93
Diegeler 2019	361	818	352	839	1.04 [0.92, 1.17]	33.42
Hueb 2010	13	142	8	145	1.60 [0.68, 3.76]	0.69
Karolak 2007	10	139	5	145	2.01 [0.71, 5.75]	0.46
Lamy 2016	346	2,029	322	2,055	1.08 [0.93, 1.24]	25.44
Puskas 2011	31	67	26	73	1.20 [0.78, 1.87]	2.60
Van Dijk 2007	10	132	6	133	1.63 [0.61, 4.37]	0.52
Quin 2022	378	726	342	757	1.10 [0.98, 1.24]	34.93
**Overall**					1.09 [1.01, 1.17]	

Heterogeneity: $\tau^2 = 0.00$, $I^2 = 0.00\%$, $H^2 = 1.00$

Test of $\theta = 0$: $z = 2.27$, $p = 0.02$

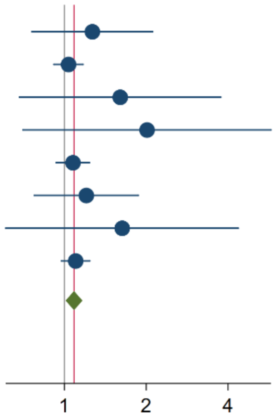

Random-effects REML model

Figure S4.3 Mid-term Stroke

Study	Off-pump CABG Events	Off-pump CABG Event-free	On-pump CABG Events	On-pump CABG Event-free	Risk ratio with 95% CI	Weight (%)
Angelini 2002	3	197	6	195	0.50 [0.13, 1.98]	4.19
Diegeler 2013	41	1,138	52	1,139	0.80 [0.53, 1.19]	48.92
Hlavicka 2016	2	96	5	102	0.44 [0.09, 2.20]	3.01
Lamy 2013	36	2,339	40	2,337	0.90 [0.58, 1.41]	39.50
Nathoe 2003	1	141	2	137	0.49 [0.04, 5.34]	1.38
Nogueira 2008	0	105	3	94	0.13 [0.01, 2.52]	0.91
Puskas 2004	2	88	2	93	1.06 [0.15, 7.34]	2.10
**Overall**					0.79 [0.60, 1.05]	

Heterogeneity: $\tau^2 = 0.00$, $I^2 = 0.00\%$, $H^2 = 1.00$

Test of $\theta = 0$: $z = -1.63$, $p = 0.10$

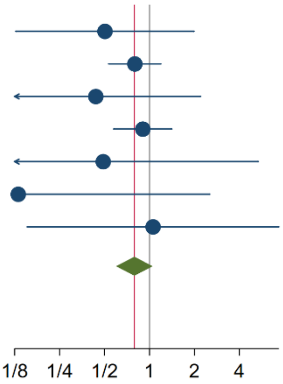

Random-effects REML model

Figure S4.4 Long-term Stroke

Study	Off-pump CABG Events	Off-pump CABG Event-free	On-pump CABG Events	On-pump CABG Event-free	Risk ratio with 95% CI	Weight (%)
Hueb 2010	3	152	5	148	0.59 [0.14, 2.44]	5.63
Lamy 2016	55	2,320	66	2,311	0.83 [0.59, 1.19]	90.10
Van Dijk 2007	2	140	5	134	0.39 [0.08, 1.98]	4.27
**Overall**					0.79 [0.57, 1.11]	

Heterogeneity: $\tau^2 = 0.00$, $I^2 = 0.00\%$, $H^2 = 1.00$

Test of $\theta = 0$: $z = -1.36$, $p = 0.17$

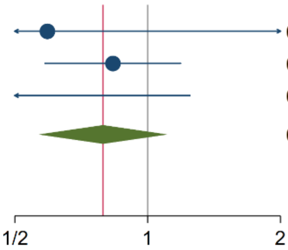

Random-effects REML model

Figure S4.5 Short-term Mortality

Study	Off-pump CABG Events	Off-pump CABG Events-free	On-pump CABG Events	On-pump CABG Events-free	Odds ratio with 95% CI	Weight (%)
Angelini 2002	0	200	2	199	0.20 (0.01, 4.17)	0.55
Chen 2004	1	149	2	148	0.50 (0.04, 5.54)	0.87
Diegeler 2013	31	1,156	34	1,173	0.93 (0.56, 1.52)	20.75
Gerola 2004	1	79	3	77	0.32 (0.03, 3.19)	0.97
Hernandez 2007	0	99	1	101	0.34 (0.01, 8.45)	0.49
Hlavicka 2016	4	94	6	101	0.72 (0.20, 2.62)	3.01
Houlind 2012	7	443	8	442	0.87 (0.31, 2.43)	4.83
Hueb 2010	1	154	0	153	2.98 (0.12, 73.74)	0.49
Iqbal 2014	2	98	3	97	0.66 (0.11, 4.04)	1.54
Khan 2004	0	54	0	49	0.91 (0.02, 46.64)	0.33
Kobayashi 2005	0	81	0	86	1.06 (0.02, 54.12)	0.33
Lamy 2012	60	2,315	59	2,318	1.02 (0.71, 1.47)	38.14
Legare 2004	2	148	1	149	2.01 (0.18, 22.45)	0.87
Lei 2014	2	220	7	216	0.28 (0.06, 1.37)	2.02
Lemma 2012	4	204	7	196	0.55 (0.16, 1.90)	3.26
Lingass 2004	1	59	1	59	1.00 (0.06, 16.37)	0.65
Moller 2010	6	170	11	152	0.49 (0.18, 1.35)	4.87
Motallebzadeh 2007	1	107	2	102	0.48 (0.04, 5.34)	0.87
Muneretto 2003	3	85	2	86	1.52 (0.25, 9.31)	1.53
Puskas 2003	1	97	2	97	0.50 (0.04, 5.61)	0.86
Raja 2003	0	150	9	141	0.05 (0.00, 0.86)	0.62
Shroyer 2009	18	1,086	13	1,086	1.38 (0.68, 2.84)	9.79
Sousa Uva 2010	0	73	0	74	1.01 (0.02, 51.76)	0.33
Straka 2004	4	200	2	182	1.82 (0.33, 10.05)	1.73
Van Dijk 2001	0	142	0	139	0.98 (0.02, 49.68)	0.33
**Overall**					0.89 (0.71, 1.12)	

Heterogeneity: $\tau^2 = 0.00$, $I^2 = 0.00\%$, $H^2 = 1.00$

Test of $\theta = 0$: $z = -1.00$, $p = 0.32$

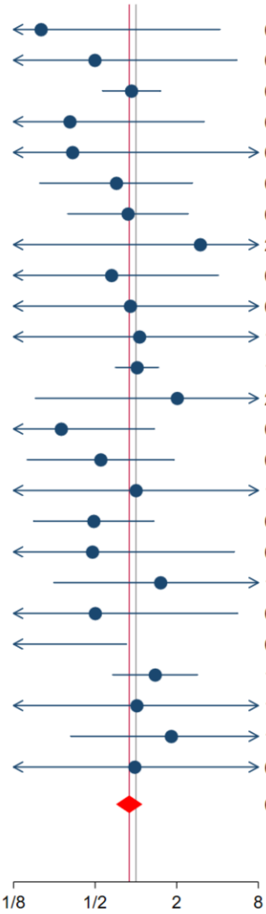

Random-effects REML model

Figure S4.6 Mid-term Mortality

Study	Off-pump CABG Events	Off-pump CABG Event-free	On-pump CABG Events	On-pump CABG Event-free	Risk ratio with 95% CI	Weight (%)
Angelini 2002	4	196	7	194	0.57 [0.17, 1.93]	1.78
Diegeler 2013	83	1,096	95	1,096	0.88 [0.66, 1.17]	32.70
Hlavicka 2016	16	82	11	96	1.59 [0.78, 3.25]	5.10
Lamy 2013	122	2,253	119	2,258	1.03 [0.80, 1.31]	43.34
Lingaas 2006	1	59	1	59	1.00 [0.06, 15.62]	0.35
Muneretto 2003	4	84	4	84	1.00 [0.26, 3.87]	1.43
Nathoe 2003	2	140	2	137	0.98 [0.14, 6.85]	0.69
Nogueira 2008	0	105	0	97	0.92 [0.02, 46.15]	0.17
Puskas 2004	4	86	4	91	1.06 [0.27, 4.09]	1.43
Shroyer 2009	43	1,018	30	1,020	1.42 [0.90, 2.24]	12.49
Sousa Uva 2010	1	72	3	71	0.34 [0.04, 3.17]	0.52
**Overall**					1.02 [0.87, 1.20]	

Heterogeneity: $\tau^2 = 0.00$, $I^2 = 0.00\%$, $H^2 = 1.00$

Test of $\theta = 0$: $z = 0.27$, $p = 0.79$

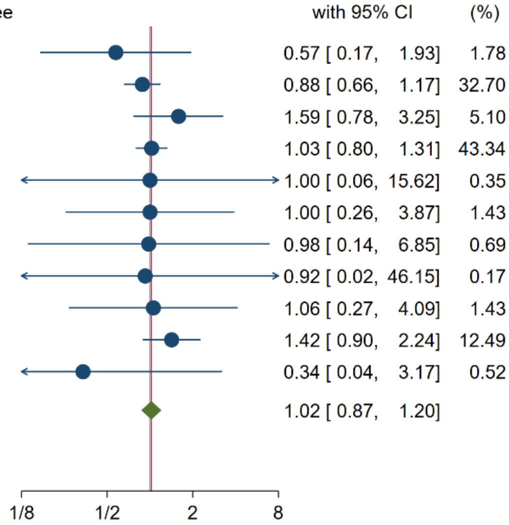

Random-effects REML model

Figure S4.7 Short-term Coronary reintervention

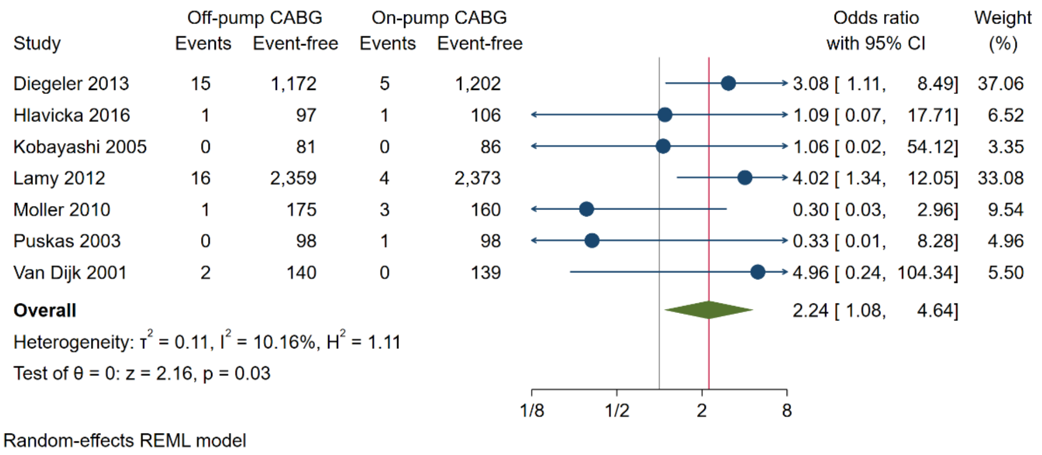

Figure S4.8 Mid-term Coronary reintervention

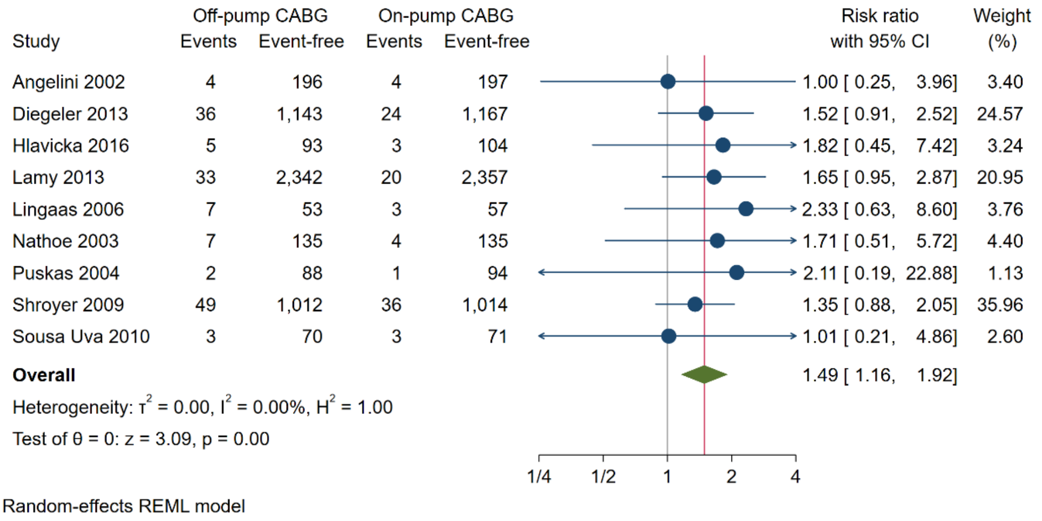

Figure S4.9 Long-term Coronary reintervention

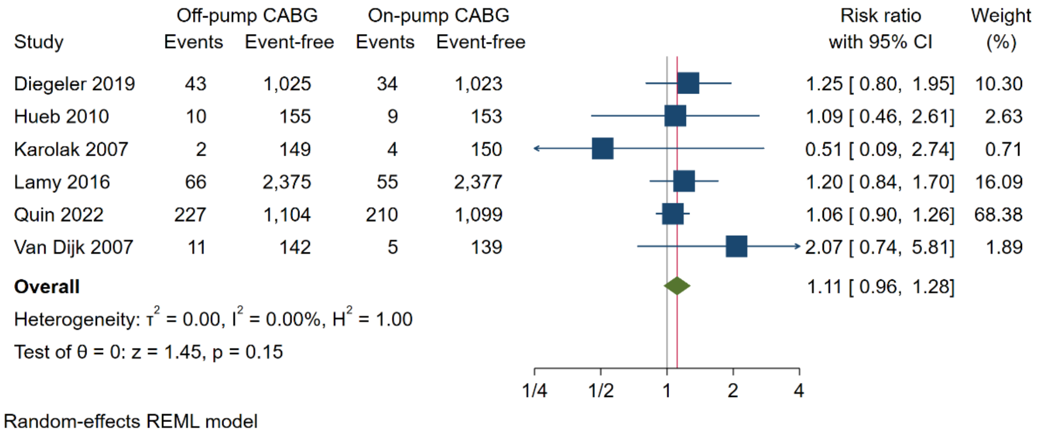

Figure S4.10 Short-term Acute renal failure requiring dialysis

Study	Off-pump CABG Events	Off-pump CABG Event-free	On-pump CABG Events	On-pump CABG Event-free	Odds ratio with 95% CI	Weight (%)
Diegeler 2013	29	1,158	37	1,170	0.79 [0.48, 1.30]	37.29
Hlavicka 2016	1	97	5	102	0.21 [0.02, 1.83]	1.93
Hueb 2010	1	154	1	152	0.99 [0.06, 15.92]	1.17
Kobayashi 2005	0	81	1	85	0.35 [0.01, 8.71]	0.88
Lamy 2012	28	2,347	27	2,350	1.04 [0.61, 1.77]	32.03
Lei 2014	2	220	5	218	0.40 [0.08, 2.06]	3.32
Moller 2010	7	169	8	155	0.80 [0.28, 2.26]	8.41
Puskas 2003	2	96	0	99	5.16 [0.24, 108.78]	0.97
Shroyer 2009	9	1,095	10	1,089	0.90 [0.36, 2.21]	11.07
Sousa Uva 2010	0	73	0	74	1.01 [0.02, 51.76]	0.59
Straka 2004	2	202	2	182	0.90 [0.13, 6.46]	2.33
**Overall**					0.85 [0.63, 1.15]	

Heterogeneity: $\tau^2 = 0.00$, $I^2 = 0.00\%$, $H^2 = 1.00$

Test of $\theta = 0$: $z = -1.06$, $p = 0.29$

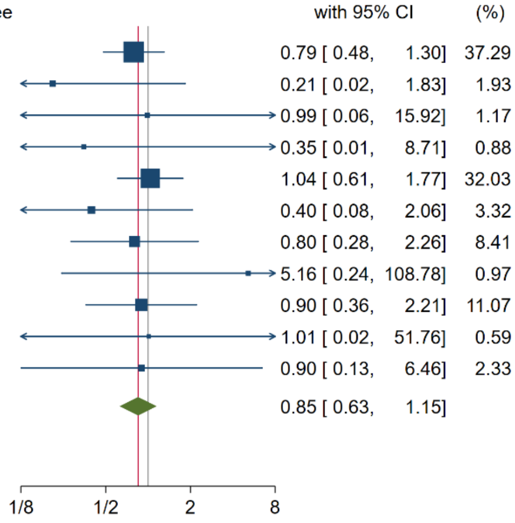

Random-effects REML model

Figure S5. Leave-one-out analysis

Figure S5.1 Short-term stroke

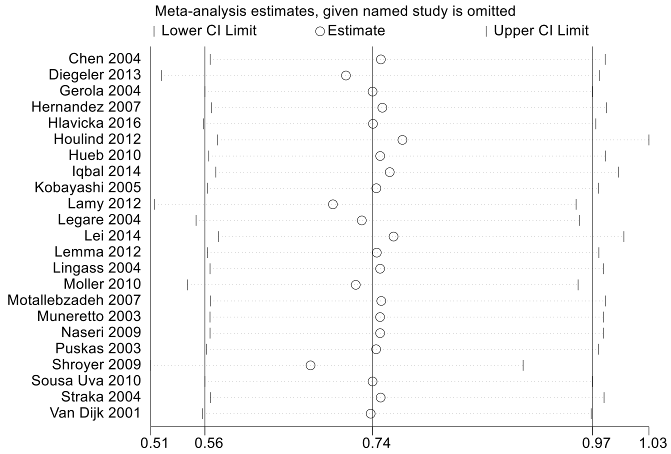

Figure S5.2 Long-term mortality

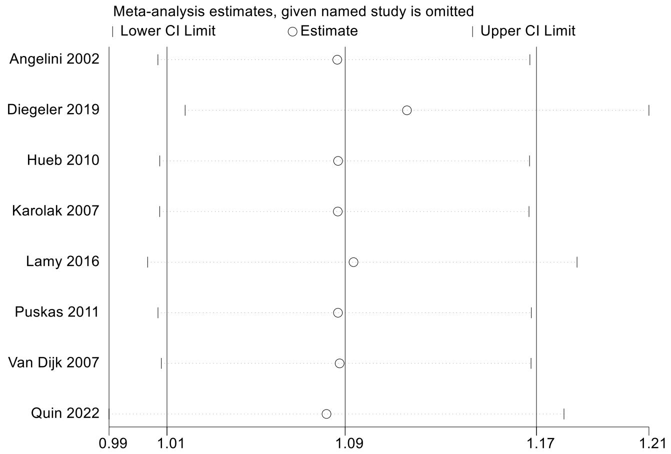

Figure S5.3 Mid-term stroke

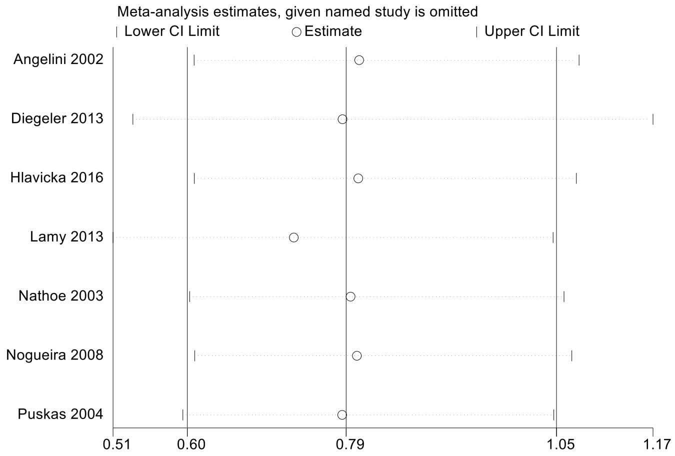

Figure S5.4 Long-term stroke

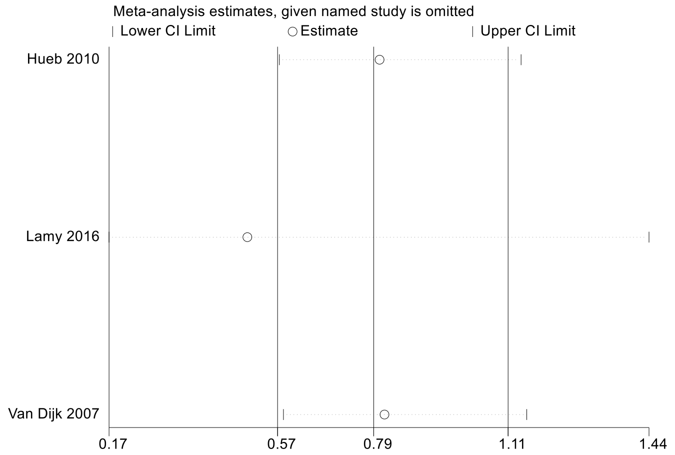

Figure S5.5 Short-term mortality

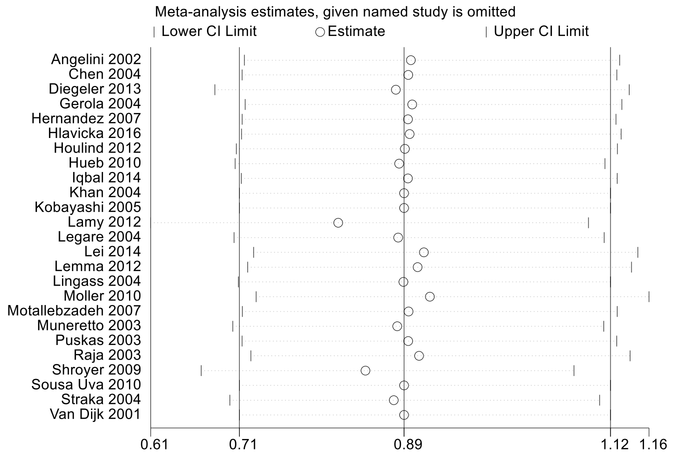

Figure S5.6 Mid-term mortality

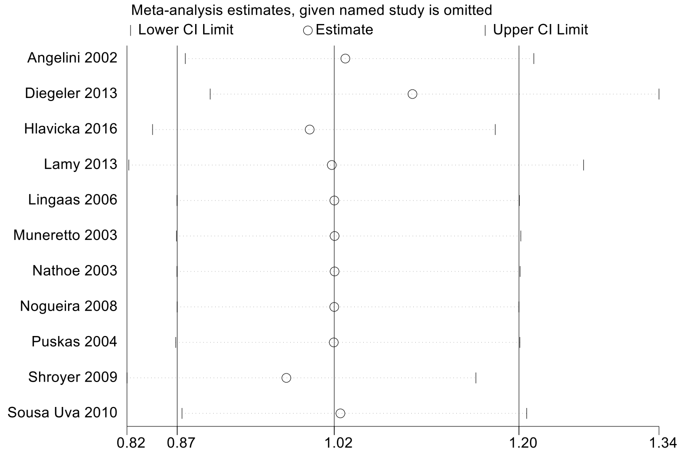

Figure S5.7 Short-term coronary reintervention

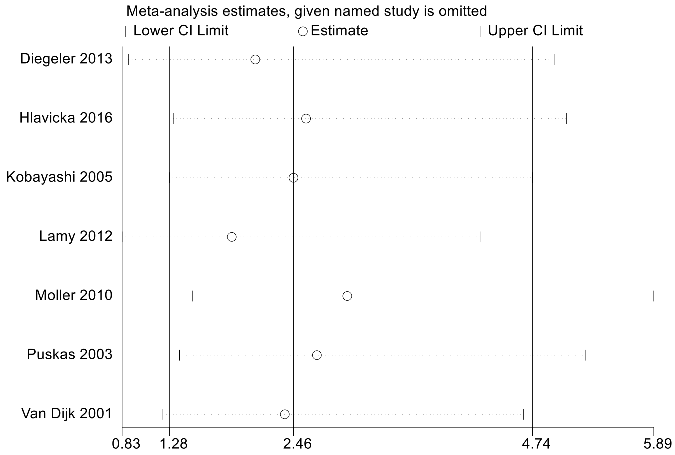

Figure S5.8 Mid-term coronary reintervention

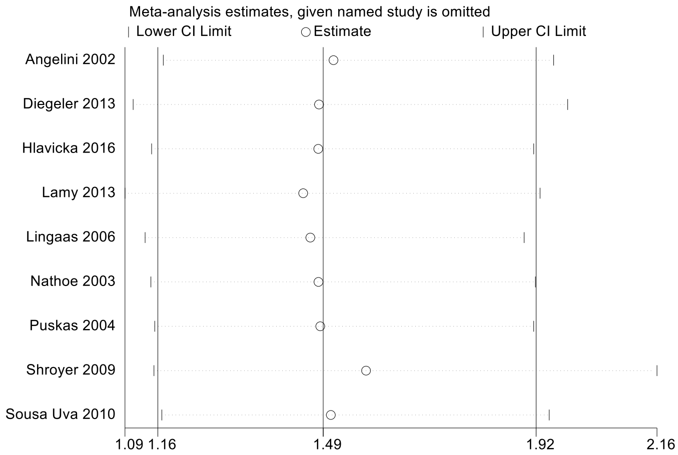

Figure S5.9 Long-term coronary reintervention

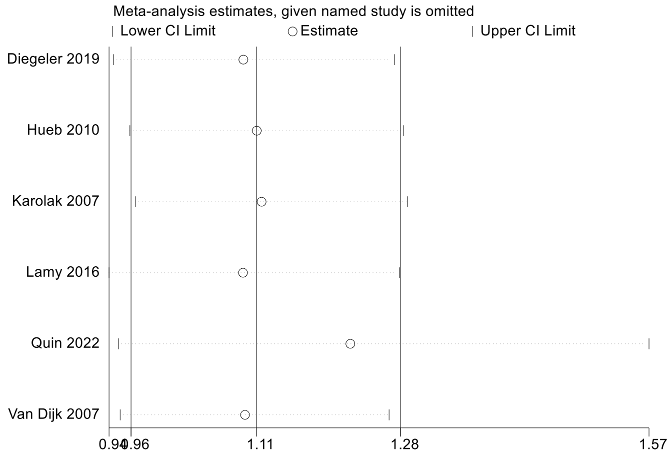

Figure S5.10 Short-term acute renal failure requiring dialysis

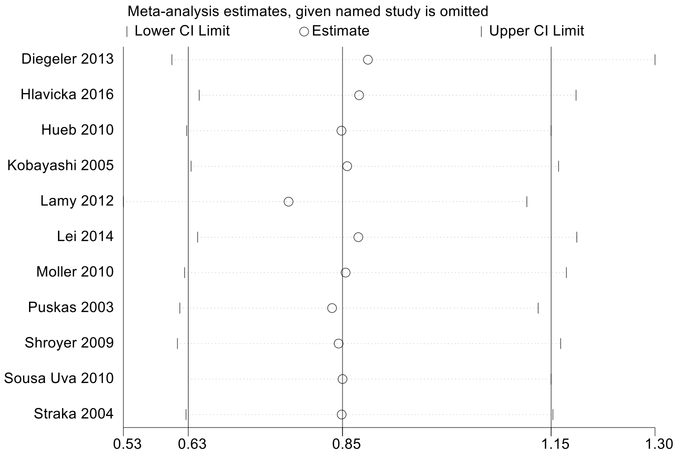

Figure S6. Sensitivity analysis

Figure S6.1 Mortality based on studies reporting 5-year Mortality

Study	Off-pump CABG Events	Off-pump CABG Event-free	On-pump CABG Events	On-pump CABG Event-free	Risk ratio with 95% CI	Weight (%)
Diegeler 2019	361	818	352	839	1.04 [0.92, 1.17]	46.45
Hueb 2010	13	142	8	145	1.60 [0.68, 3.76]	0.96
Lamy 2016	346	2,029	322	2,055	1.08 [0.93, 1.24]	35.36
Puskas 2011	7	91	18	81	0.39 [0.17, 0.90]	1.02
Shroyer 2017	168	936	131	968	1.28 [1.03, 1.58]	15.48
Van Dijk 2007	10	132	6	133	1.63 [0.61, 4.37]	0.72
**Overall**					1.08 [0.99, 1.18]	

Heterogeneity: $I^2 = 50.32\%$, $H^2 = 2.01$

Test of θ = 0: z = 1.84, p = 0.07

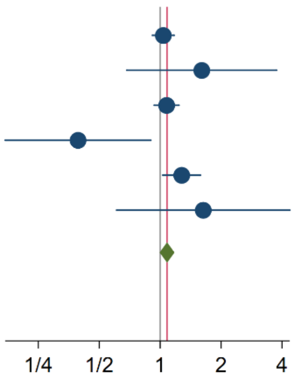

Fixed-effects inverse-variance model

This meta-analysis was conducted exclusively using studies that presented 5-year Mortality rates. The analysis utilized a Fixed-effects model along with the Inverse Variance method.

Figure S6.2 Mortality based on studies directly reporting HR

Study or Subgroup	Off-pump CABG Events	Off-pump CABG Total	On-pump CABG Events	On-pump CABG Total	O-E	Variance	Weight	Hazard Ratio Exp[(O-E) / V], Fixed, 95% CI
Angelini 2009	29	200	23	201	2.76	12.84	2.0%	1.24 [0.72, 2.14]
Diegeler 2019	361	1179	352	1191	5.38	182.1	28.1%	1.03 [0.89, 1.19]
Lamy 2016	346	2375	322	2377	21.49	279.27	43.1%	1.08 [0.96, 1.21]
Quin 2022	378	1104	342	1099	22.7	173.23	26.8%	1.14 [0.98, 1.32]
**Total (95% CI)**		**4858**		**4868**			**100.0%**	**1.08 [1.00, 1.17]**
Total events	1114		1039					

Heterogeneity: $Chi^2 = 1.15$, df = 3 (P = 0.76); $I^2 = 0\%$

Test for overall effect: Z = 2.06 (P = 0.04)

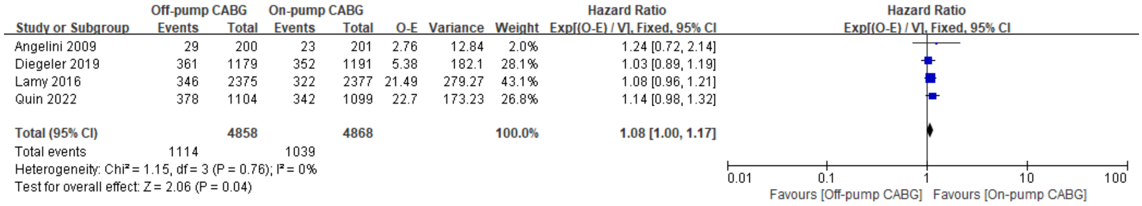

This meta-analysis was conducted solely using studies that directly reported the Hazard Ratio (HR) along with its corresponding 95% confidence interval

Figure S7. Subgroup analysis based on crossover rate

Figure S7.1 Subgroup analysis for short-term mortality

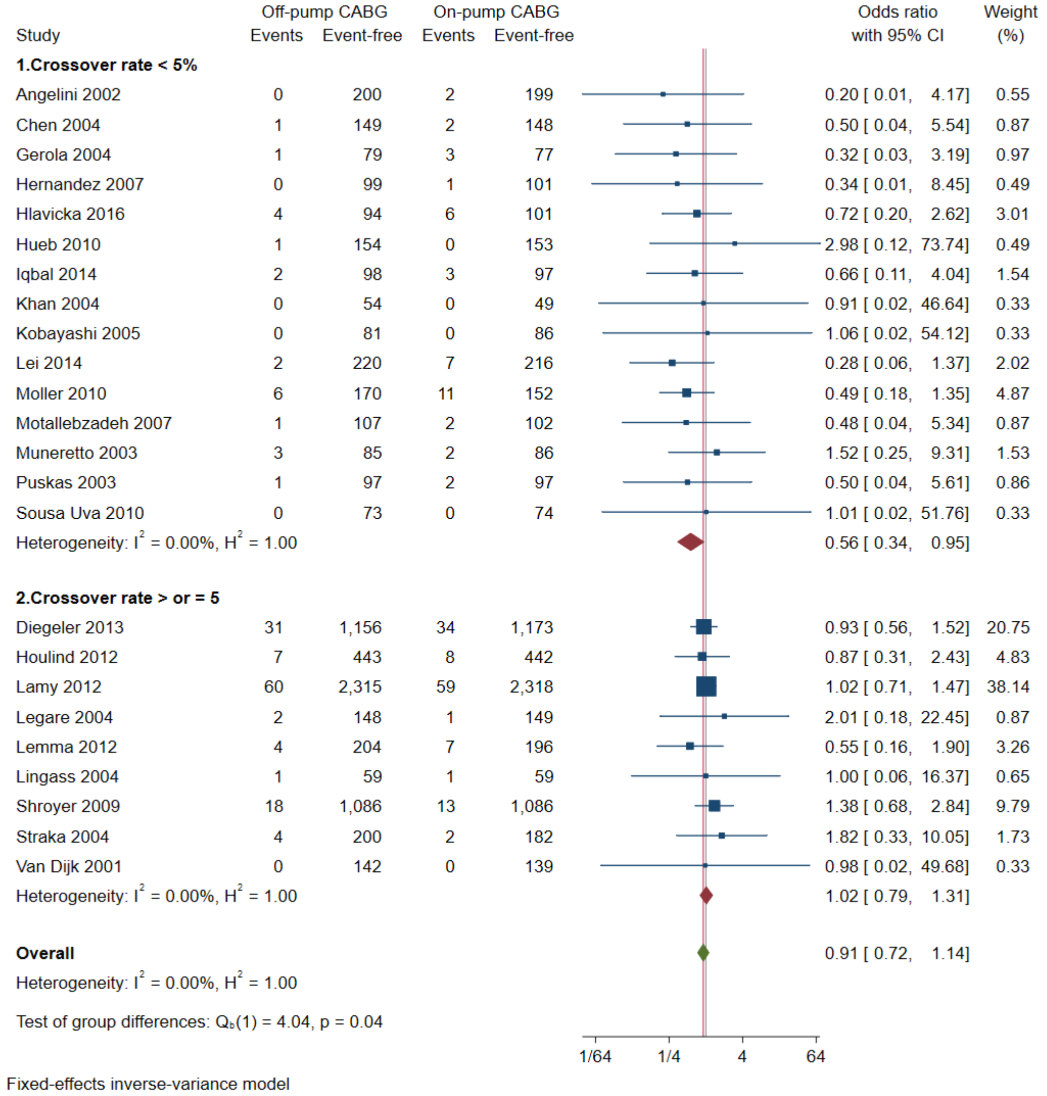

Figure S7.2 Subgroup analysis for short-term coronary reintervention

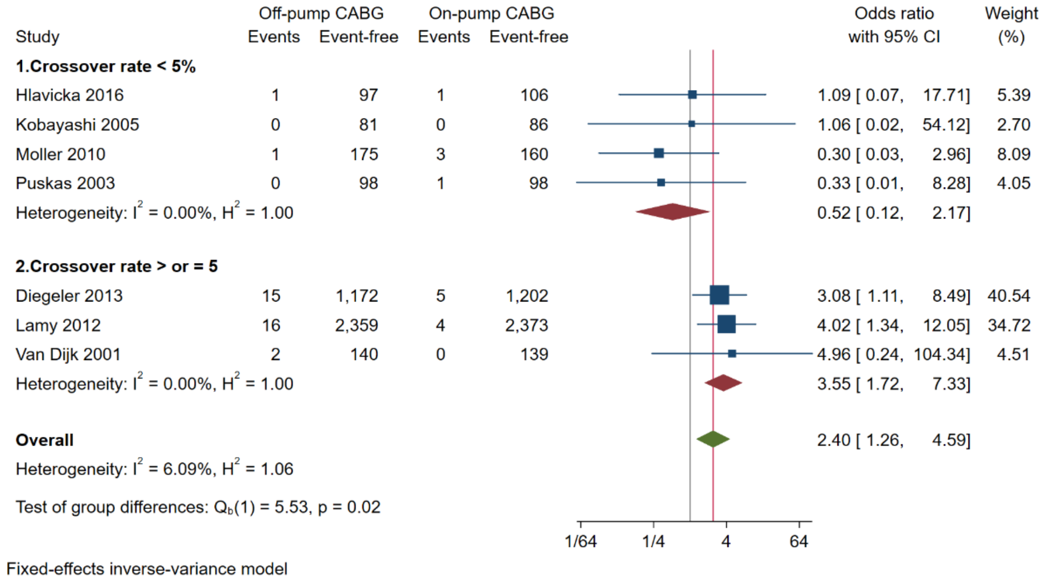

Figure S8. Trial sequential analysis

Figure S8.1 Short-term mortality

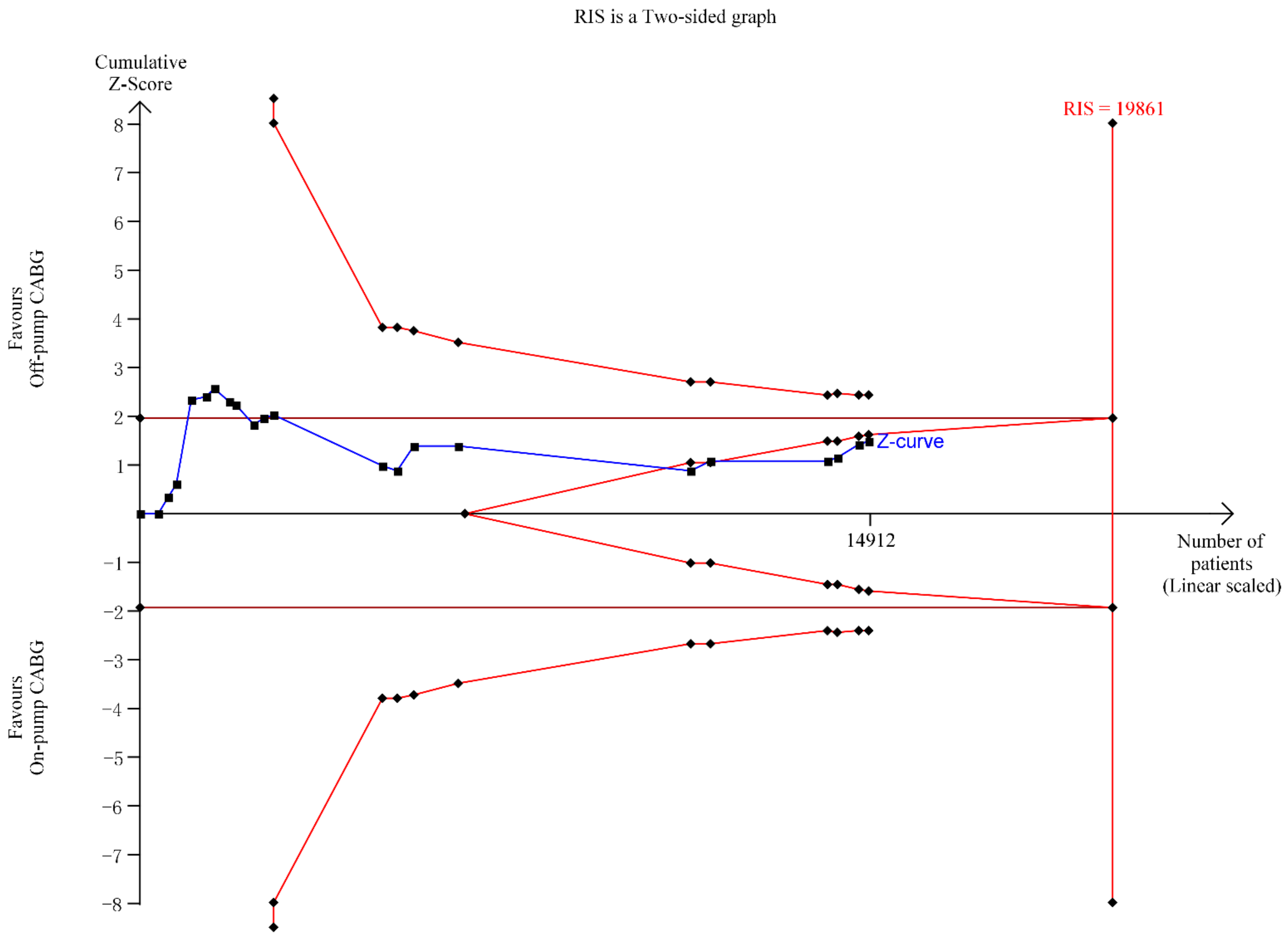

The heterogeneity-adjusted requires information size (RIS) to demonstrate or reject an intervention effect of 25% relative risk reduction of short-term mortality, occurrence of short-term mortality in the on-pump CABG of 2.15%, alpha = 5% and beta = 20% is 19861 patients. Although cumulative curve does not reach the RIS, it has crossed an invalid boundary before reaching the RIS. Therefore, it is conclusive that there exists no significant difference in short-term mortality between off-pump CABG and on-pump CABG.

Figure S8.2 Short-term stroke

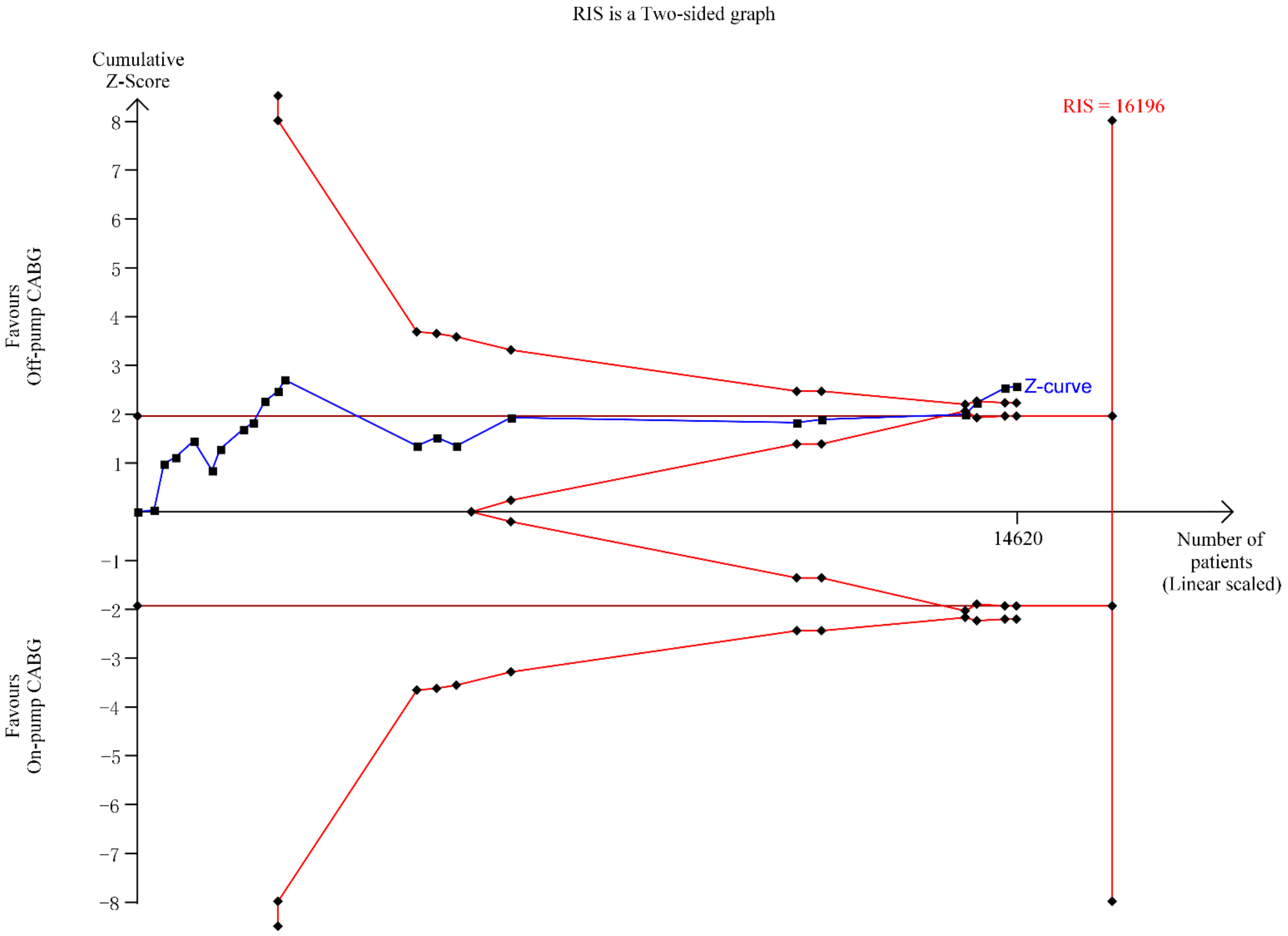

The heterogeneity-adjusted requires information size (RIS) to demonstrate or reject an intervention effect of 25% relative risk reduction of short-term stroke, occurrence of short-term stroke in the on-pump CABG of 2.63%, alpha = 5% and beta = 20% is 16196 patients. Although cumulative curve does not reach the RIS, it has crossed a sequential monitoring boundary before reaching the RIS. Therefore, it is conclusive that the incidence of short-term stroke in off-pump CABG is significantly lower than in on-pump CABG.

Figure S8.3 Short-term coronary reintervention

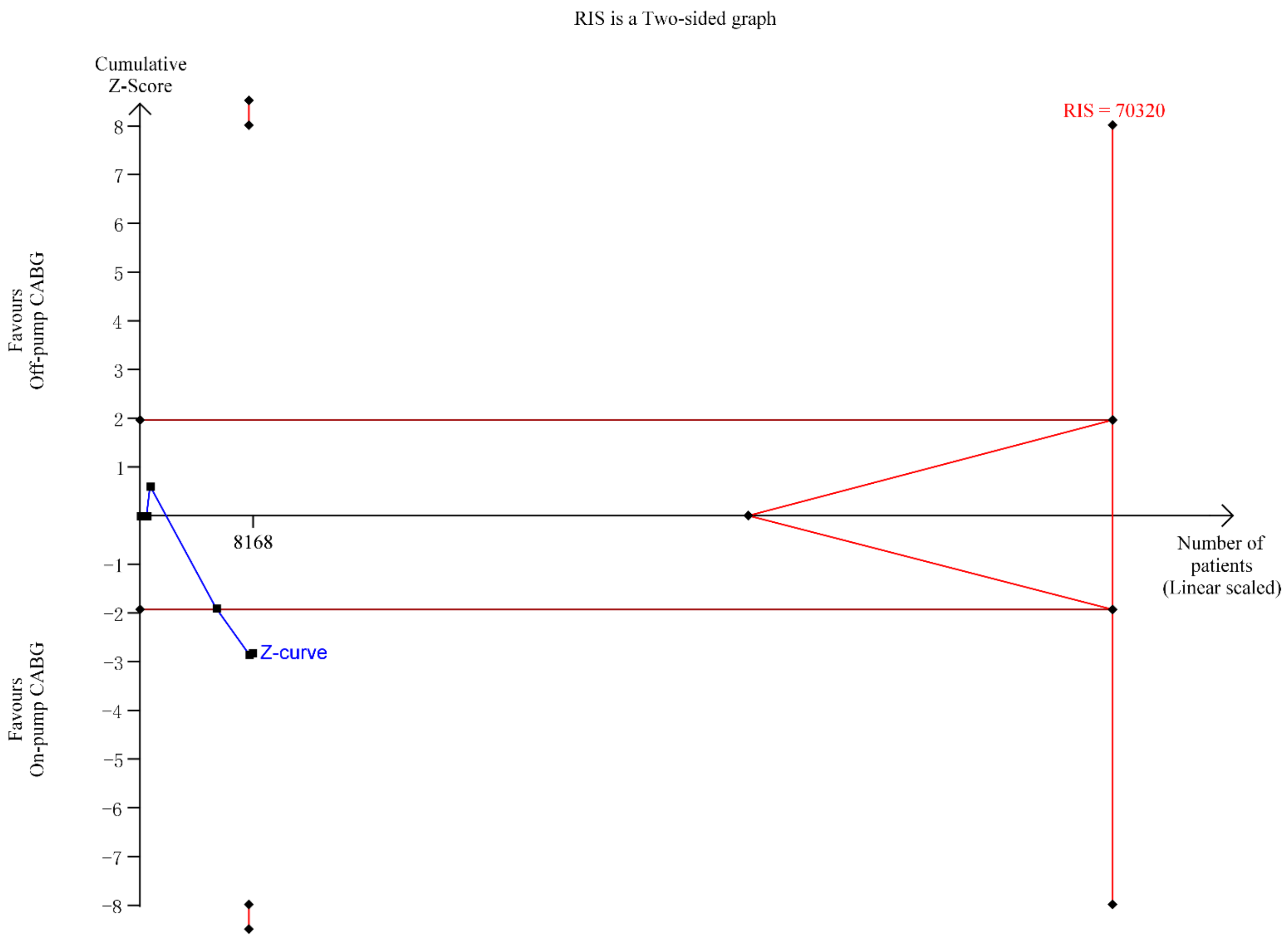

The heterogeneity-adjusted requires information size (RIS) to demonstrate or reject an intervention effect of 25% relative risk reduction of short-term coronary reintervention, occurrence of short-term coronary reintervention in the on-pump CABG of 0.93%, alpha = 5% and beta = 20% is 70320 patients. Although cumulative curve has crossed conventionally significant boundary, it does not crosse a sequential monitoring boundary before reaching the RIS. Therefore, the increasing incidence of short-term coronary reintervention associated with off-pump CABG may be a false positive effect. This conclusion still requires more trials to verify.

Figure S8.4 Short-term acute renal failure requiring dialysis

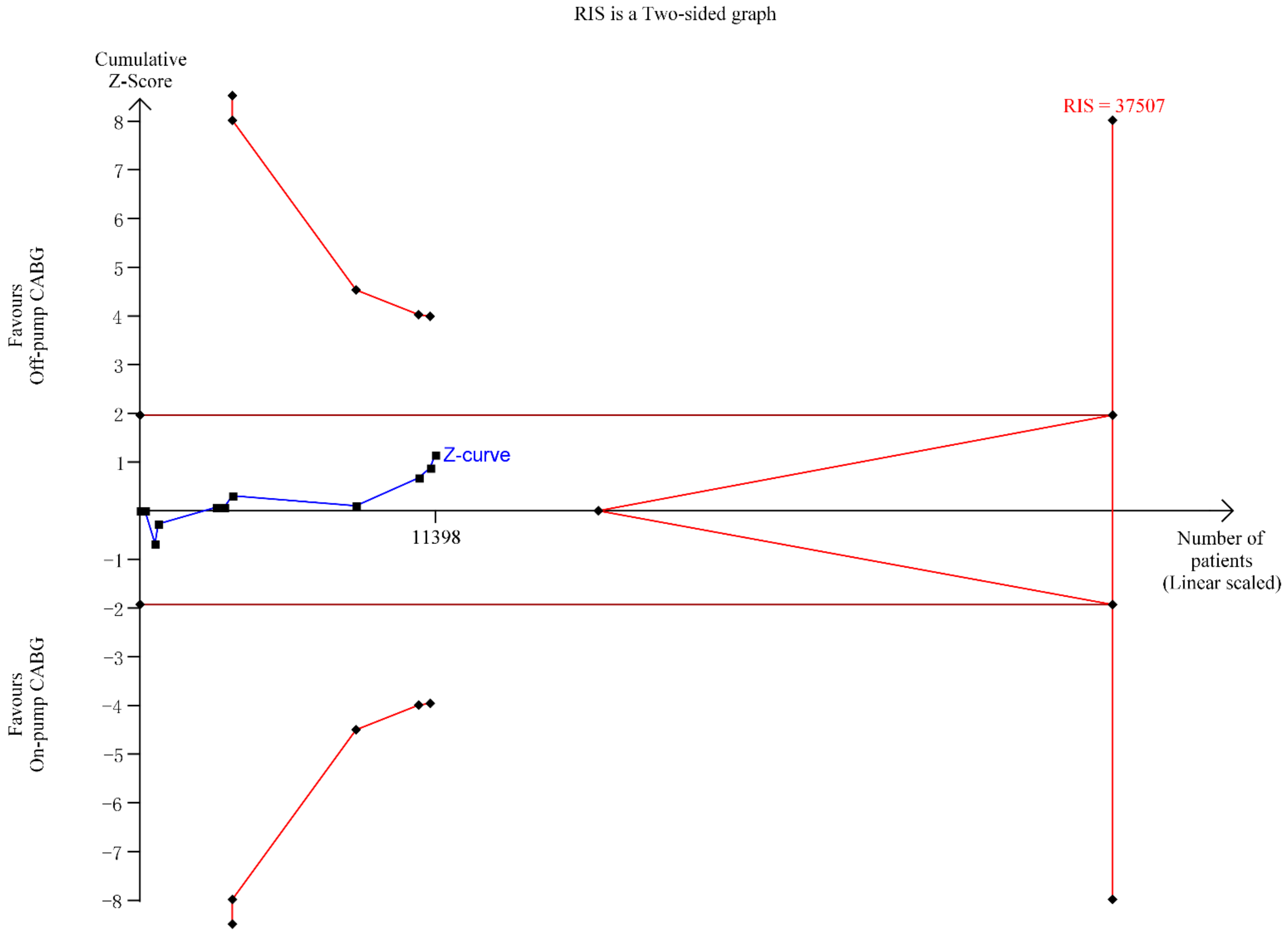

The heterogeneity-adjusted required information size (RIS) to demonstrate or reject an intervention effect of 25% relative risk reduction of short-term acute renal failure requiring dialysis, occurrence of short-term acute renal failure requiring dialysis in the on-pump CABG of 1.16%, alpha = 5% and beta = 20% is 37507 patients. The cumulative curve neither crosses a sequential monitoring boundary nor an invalid boundary before reaching the RIS. Additionally, it does not reach the RIS. Therefore, there may be not difference for the incidence of short-term acute renal failure requiring dialysis between on-pump CABG and off-pump CABG. However, this conclusion still requires more trials to verify.

Figure S8.5 Mid-term mortality

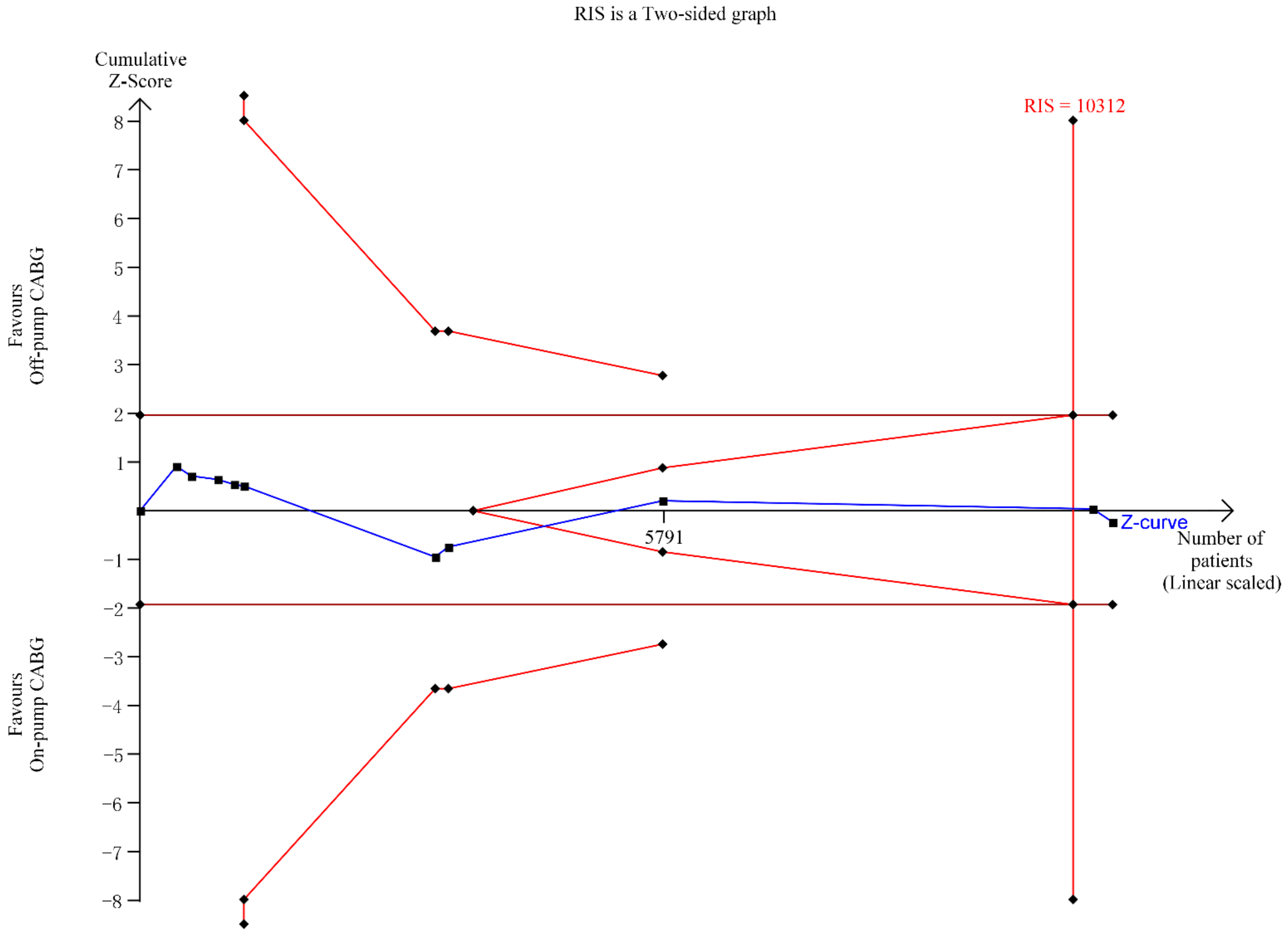

The heterogeneity-adjusted requires information size (RIS) to demonstrate or rejection an intervention effect of 25% relative risk reduction of mid-term mortality, occurrence of mid-term mortality in the on-pump CABG of 4.13%, alpha = 5% and beta = 20% is 10312 patients. The cumulative curve not only crosses an invalid boundary before reaching the RIS but also reaches the RIS. Therefore, it is conclusive that there is no significant difference in mid-term mortality between off-pump CABG and on-pump CABG.

Figure S8.6 Mid-term stroke

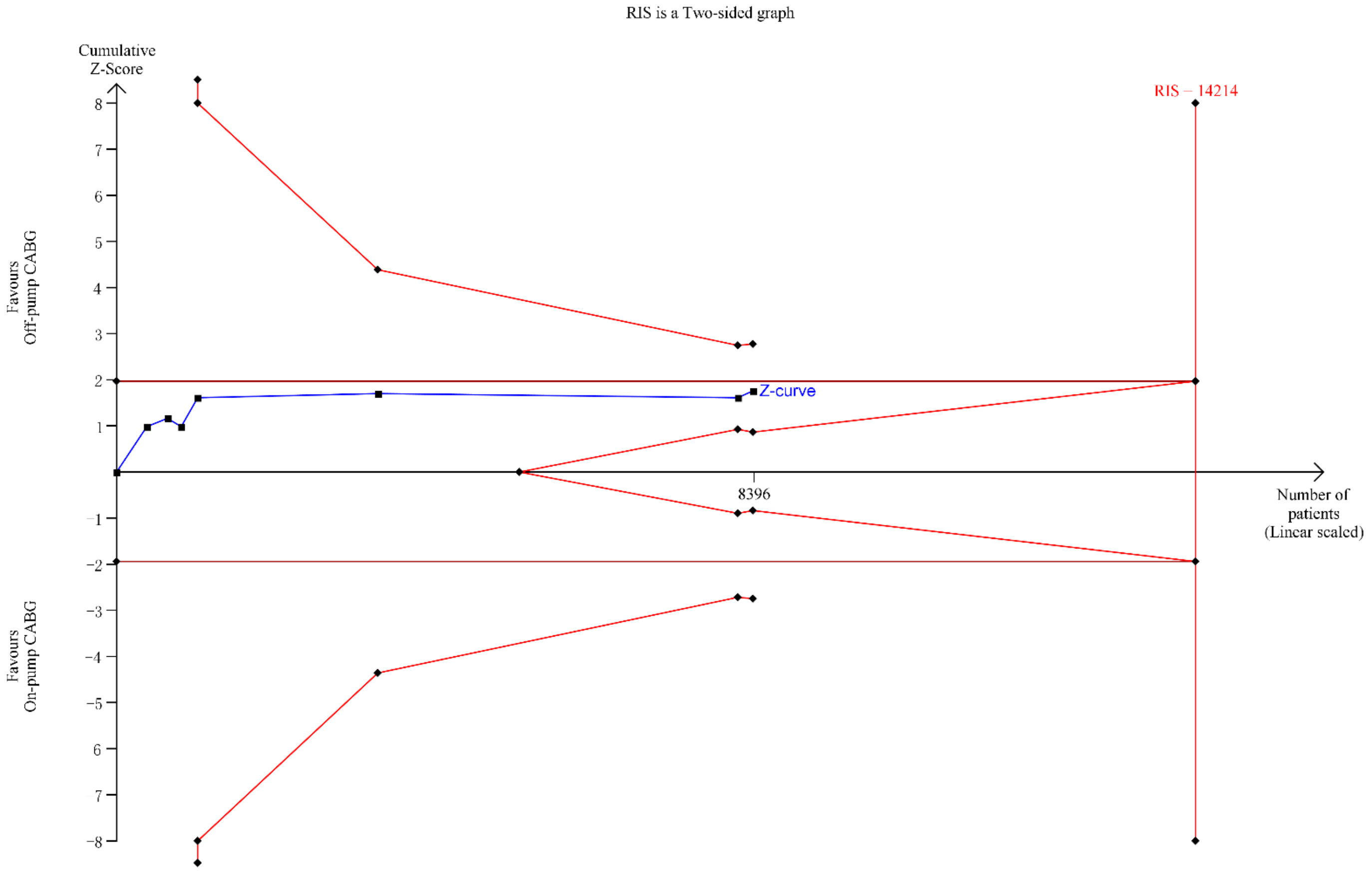

The heterogeneity-adjusted required information size (RIS) to demonstrate or reject an intervention effect of 25% relative risk reduction of mid-term stroke, occurrence of mid-term stroke in the off-pump CABG of 2.99%, alpha = 5% and beta = 20% is 14214 patients. The cumulative curve neither crosses a sequential monitoring boundary nor an invalid boundary before reaching the RIS. Additionally, it does not reach the RIS. Therefore, there may be no difference for the incidence of mid-term stroke between on-pump CABG and off-pump CABG. However, this conclusion still requires more trials to verify.

Figure S8.7 Mid-term coronary reintervention

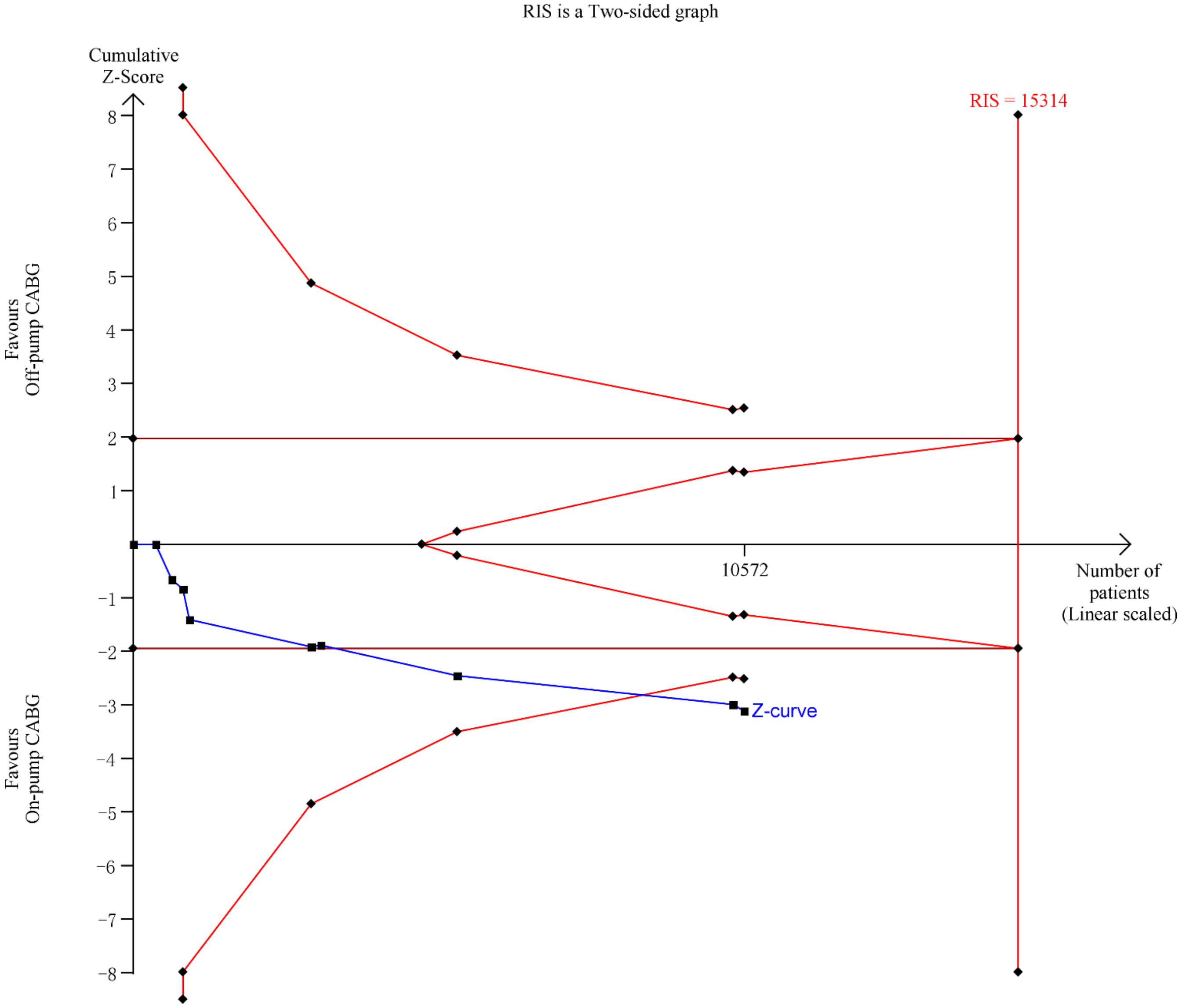

The heterogeneity-adjusted requires information size (RIS) to demonstrate or reject an intervention effect of 25% relative risk reduction of mid-term coronary reintervention, occurrence of mid-term coronary reintervention in the on-pump CABG of 2.80%, alpha = 5% and beta = 20% is 15314 patients. Although cumulative curve does not reach the RIS, it has crossed a sequential monitoring boundary before reaching the RIS. Therefore, it is conclusive that the incidence of mid-term coronary reintervention in off-pump CABG is significantly higher than in on-pump CABG.

Figure S8.8 Long-term mortality

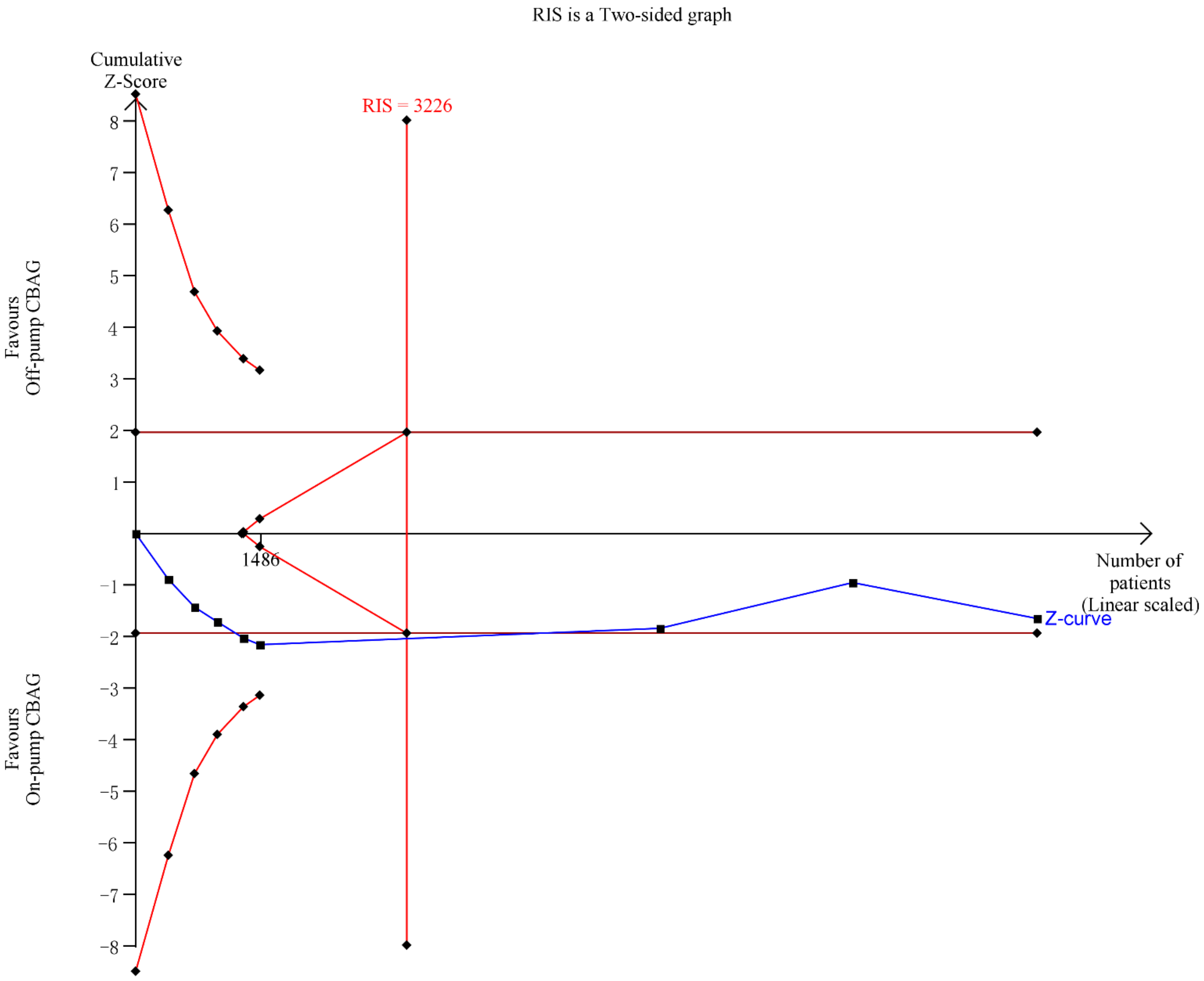

The heterogeneity-adjusted requires information size (RIS) to demonstrate or reject an intervention effect of 25% relative risk reduction of long-term mortality, occurrence of long-term mortality in the on-pump CABG of 12.49%, alpha = 5% and beta = 20% is 3226 patients. When the cumulative curve reaches the RIS, it crosses the conventionally significant boundary but does not cross sequential monitoring boundary. Therefore, there may be a higher long-term mortality rates associated with off-pump CABG compared to on-pump CABG. This conclusion still requires more trials to verify.

Figure S8.9 Long-term stroke

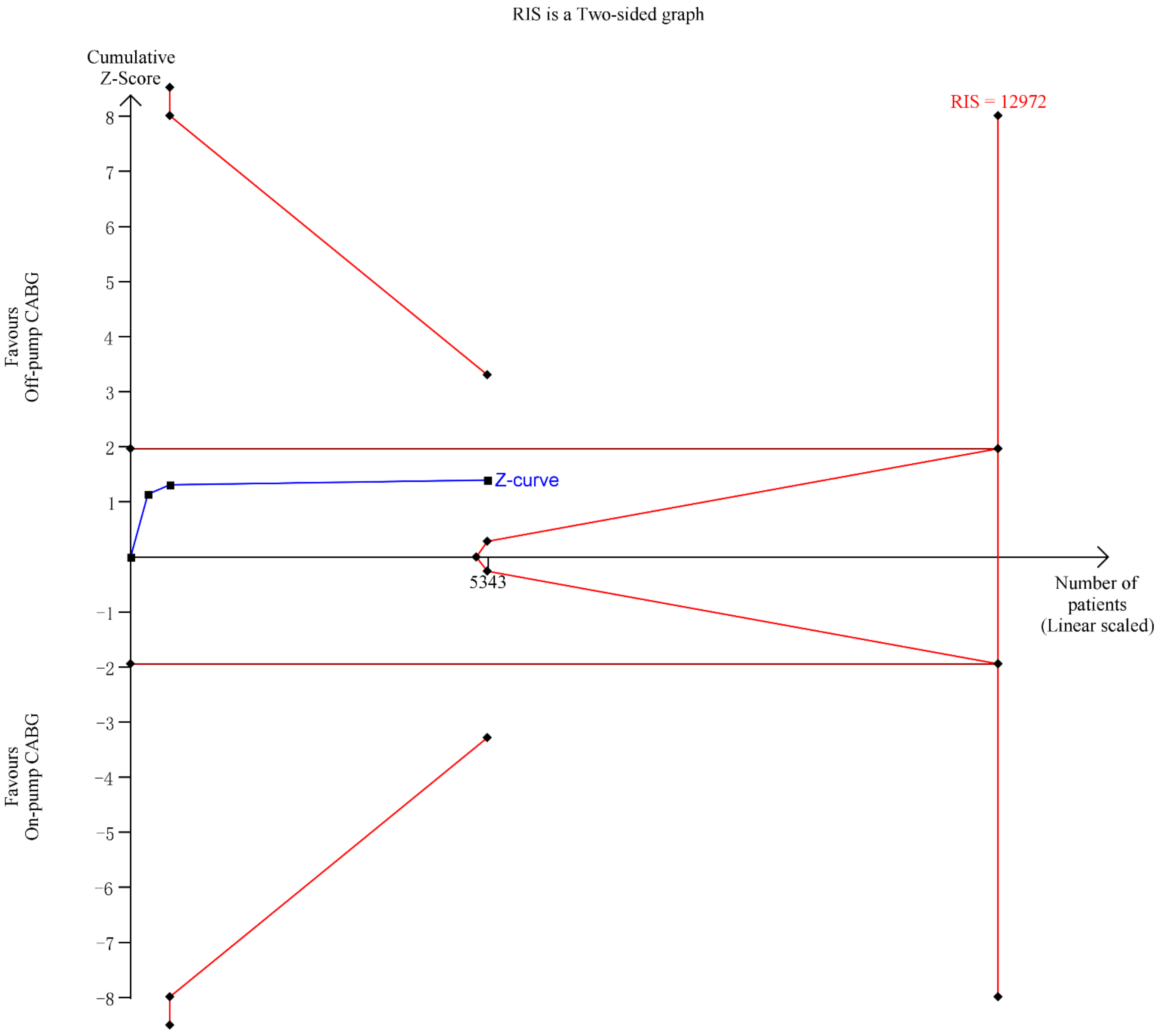

The heterogeneity-adjusted required information size (RIS) to demonstrate or reject an intervention effect of 25% relative risk reduction of long-term stroke, occurrence of long-term stroke in the off-pump CABG of 3.27%, alpha = 5% and beta = 20% is 12972 patients. The cumulative curve neither crosses a sequential monitoring boundary nor an invalid boundary before reaching the RIS. Additionally, it does not reach the RIS. Therefore, there may be no difference for the incidence of long-term stroke between on-pump CABG and off-pump CABG. However, this conclusion still requires more trials to verify.

Figure S8.10 Long-term coronary reintervention

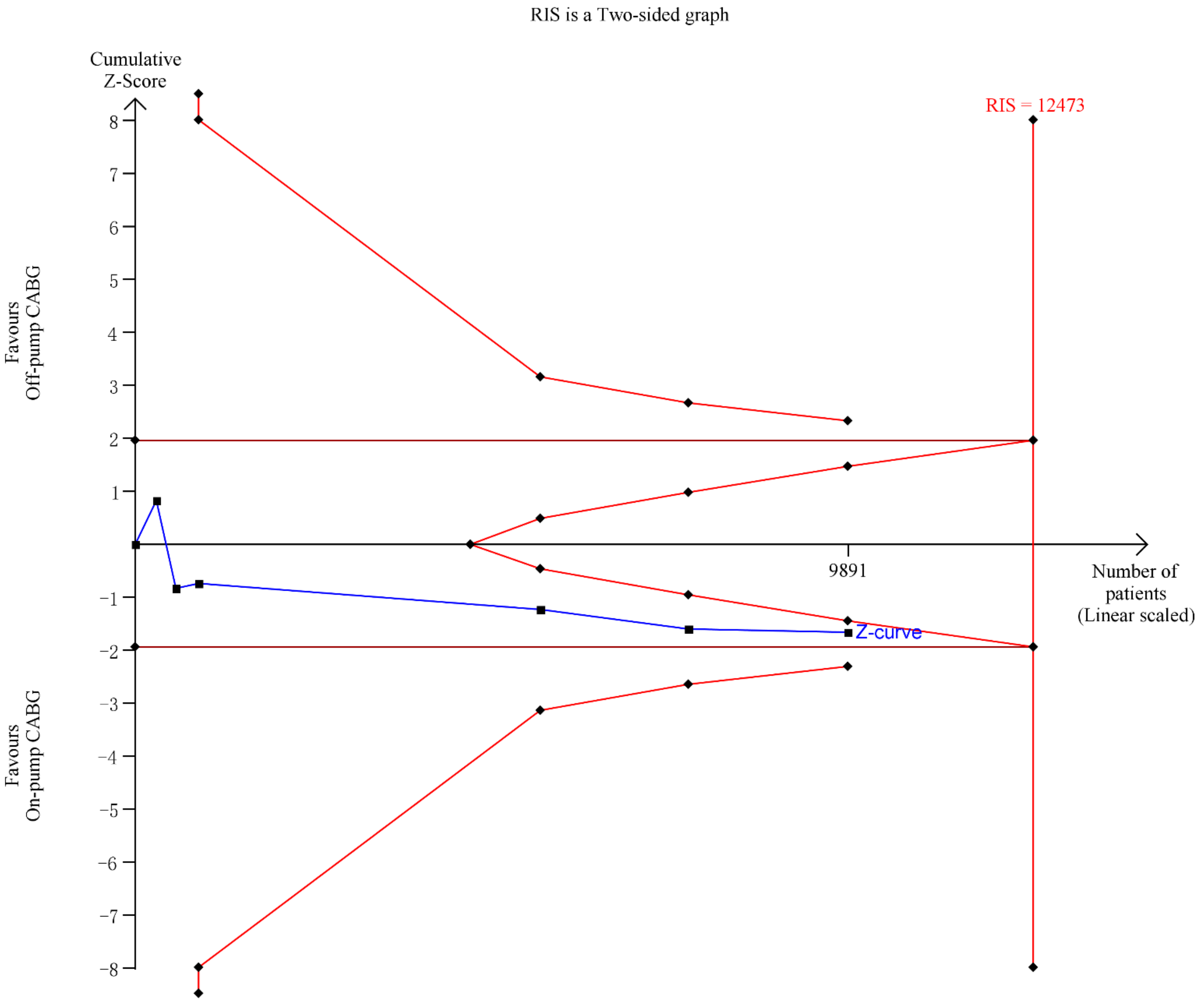

The heterogeneity-adjusted required information size (RIS) to demonstrate or reject an intervention effect of 25% relative risk reduction of long-term coronary reintervention, occurrence of long-term coronary intervention in the off-pump CABG of 3.46%, alpha = 5% and beta = 20% is 12473 patients. The cumulative curve neither crosses a sequential monitoring boundary nor an invalid boundary before reaching the RIS. Additionally, it does not reach the RIS. Therefore, there may be no difference for long-term coronary reintervention between on-pump CABG and off-pump CABG. However, this conclusion still requires more trials to verify.

Figure S9. Study-specific risk of bias

Figure S9.1 Risk of bias graph in study of report short-term outcome

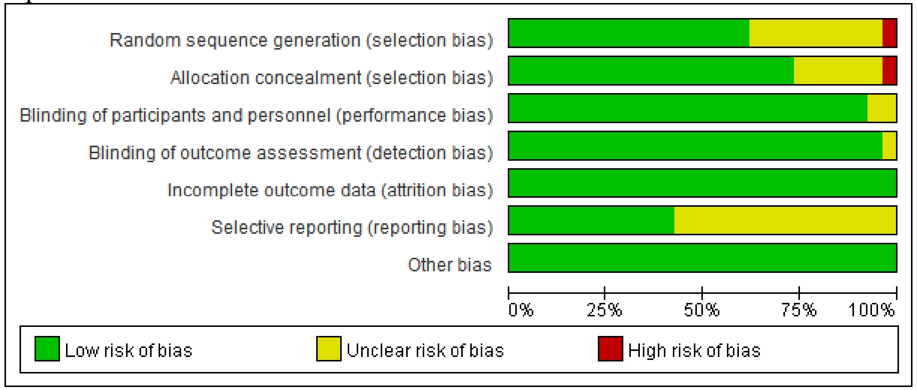

Figure S9.2 Risk of bias summary in study of report short-term outcome

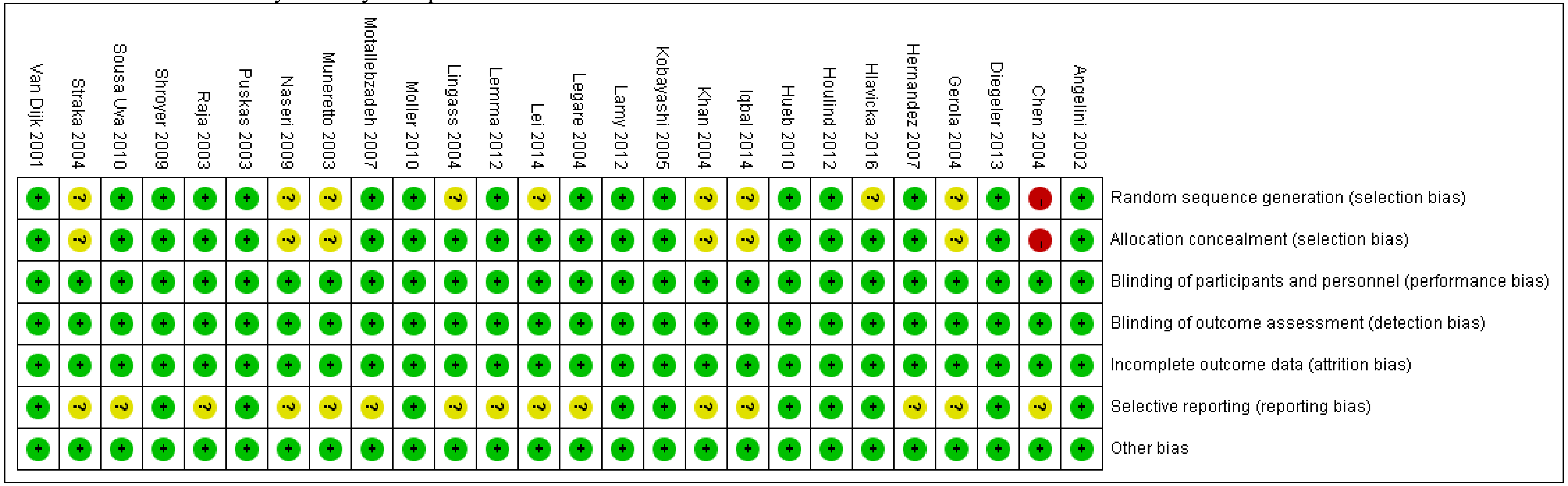

Figure S9.3 Risk of bias graph in study of report mid-term outcome

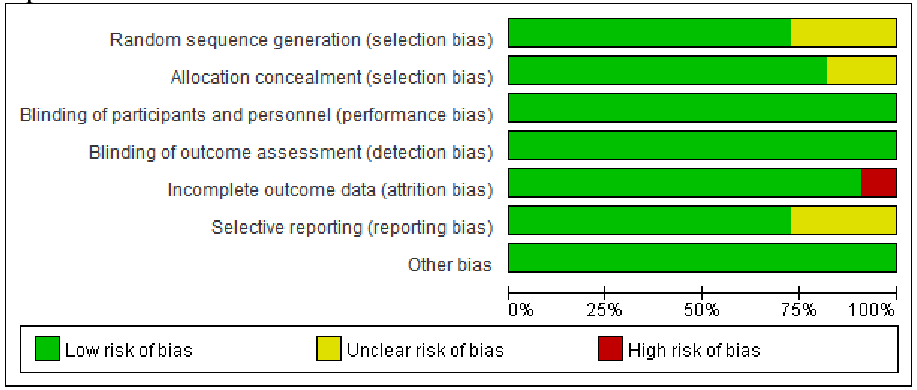

Figure S9.4 Risk of bias summary in study of report mid-term outcome

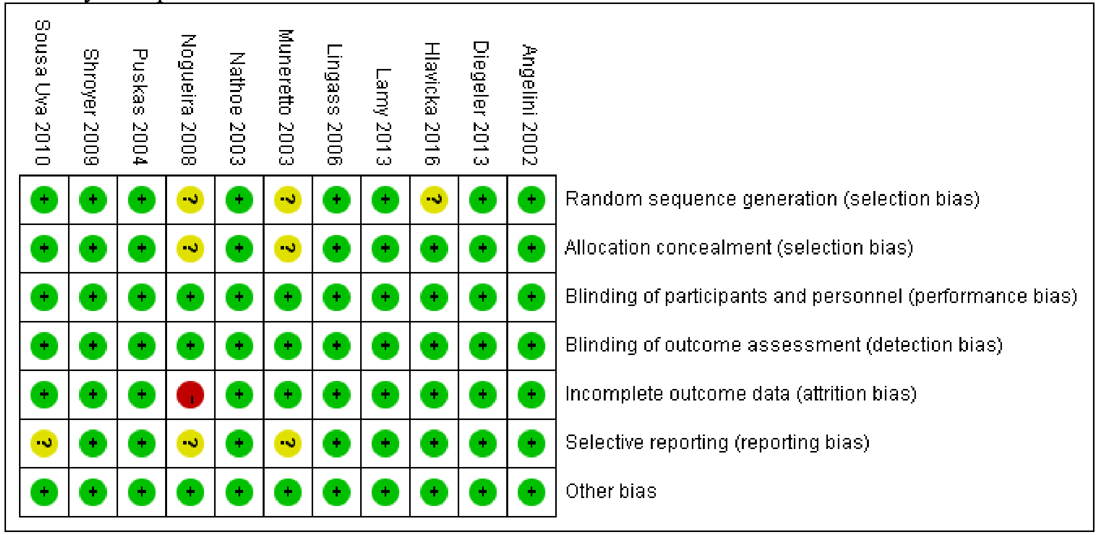

Figure S9.5 Risk of bias graph in study of report long-term outcome

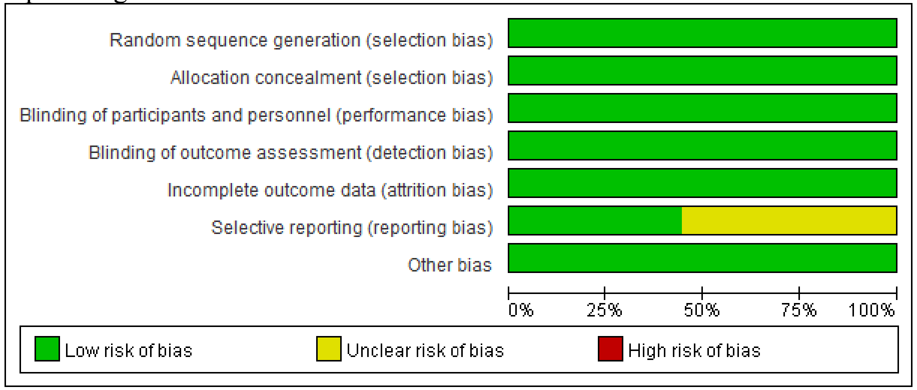

Figure S9.6 Risk of bias summary in study of report long-term outcome

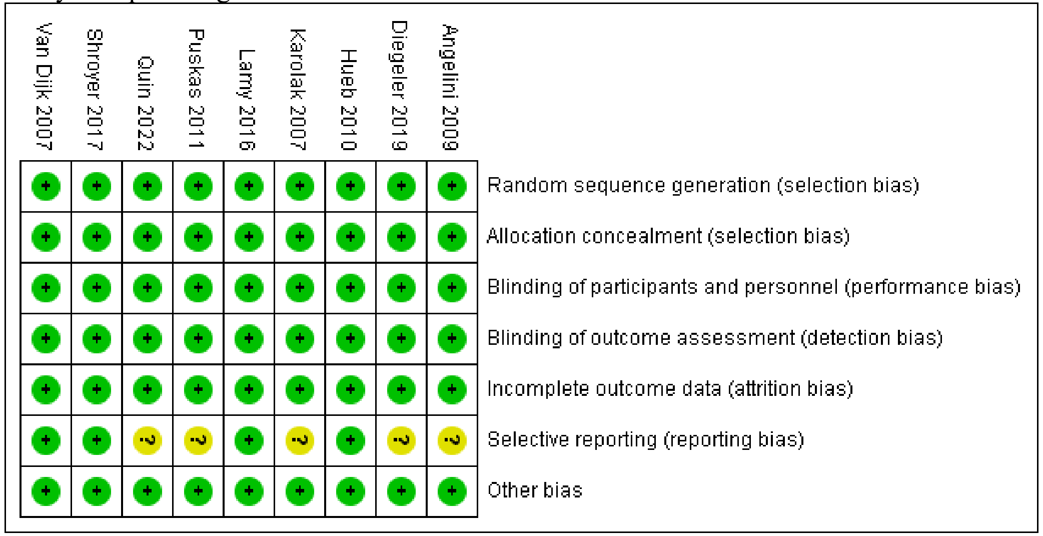

Figure S10. Funnel plot

Figure S10.1 Funnel plot in short-term mortality

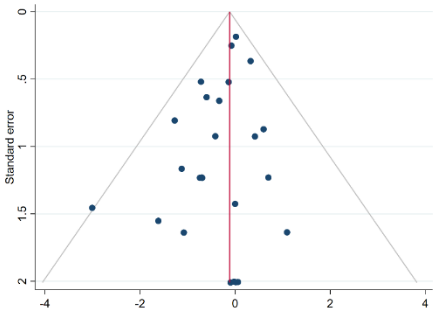

Figure S10.2 Funnel plot in short-term stroke

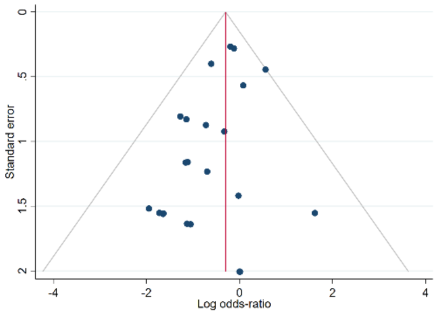

Figure S10.3 Funnel plot in short-term acute renal failure requiring dialysis

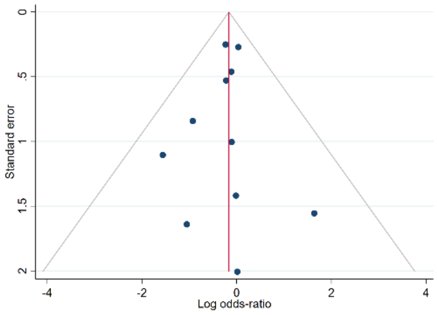

Figure S10.4 Funnel plot in mid-term Mortality

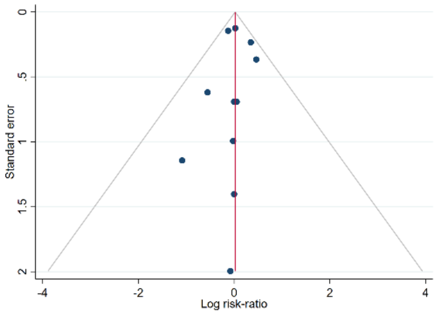

Figure S11. GRADE evidence of profile and summary of Findings table

Figure S11.1 GRADE evidence of profile of short-term outcomes

Certainty assessment							Summary of findings				
Participants (studies) Follow-up	Risk of bias	Inconsistency	Indirectness	Imprecision	Publication bias	Overall certainty of evidence	Study event rates (%)		Relative effect (95% CI)	Anticipated absolute effects	
							With On-pump	With Off-pump		Risk with On-pump	Risk difference with Off-pump
**Short-term All-cause mortality**											
15610 (25 RCTs)	not serious	not serious	not serious	not serious	none	⊕⊕⊕⊕ High	175/7798 (2.2%)	149/7812 (1.9%)	**OR 0.89** (0.71 to 1.12)	22 per 1,000	**2 fewer per 1,000** (from 6 fewer to 3 more)
**Short-term Stroke**											
14926 (23 RCTs)	not serious	not serious	not serious	not serious	none	⊕⊕⊕⊕ High	133/7458 (1.8%)	95/7468 (1.3%)	**OR 0.74** (0.57 to 0.97)	18 per 1,000	**5 fewer per 1,000** (from 8 fewer to 1 fewer)
**Short-term Coronary reintervention**											
8335 (7 RCTs)	not serious	not serious	not serious	serious[a]	none	⊕⊕⊕◯ Moderate	14/4178 (0.3%)	35/4157 (0.8%)	**OR 2.40** (1.26 to 4.59)	3 per 1,000	**5 more per 1,000** (from 1 more to 12 more)
**Short-term Acute renal failure requiring dialysis**											
11545 (11 RCTs)	not serious	not serious	not serious	serious[b]	none	⊕⊕⊕◯ Moderate	96/5772 (1.7%)	81/5773 (1.4%)	**OR 0.85** (0.63 to 1.15)	17 per 1,000	**2 fewer per 1,000** (from 6 fewer to 2 more)

CI: confidence interval; **OR:** odds ratio; **RR:** risk ratio

Explanations

a. In Trial sequential analysis, while the cumulative curve has crossed the conventionally significant boundary, it does not cross a sequential monitoring boundary before reaching the RIS. Therefore, there may exist a false positive effect. Further trials are needed to confirm this conclusion.

b. In Trial sequential analysis, the cumulative curve neither crosses a sequential monitoring boundary nor an invalid boundary before reaching the RIS. Additionally, it does not reach the RIS. Therefore, there may be no difference for the incidence of this outcome between on-pump CABG and

Figure S11.2 GRADE evidence of profile of mid-term outcomes

Certainty assessment							Summary of findings				
Participants (studies) Follow-up	Risk of bias	Inconsistency	Indirectness	Imprecision	Publication bias	Overall certainty of evidence	Study event rates (%)		Relative effect (95% CI)	Anticipated absolute effects	
							With On-pump	With Off-pump		Risk with On-pump	Risk difference with Off pump
**Mid-term All-cause mortality**											
10950 (11 RCTs)	not serious	not serious	not serious	not serious	none	⊕⊕⊕⊕ High	276/5479 (5.0%)	280/5471 (5.1%)	**RR 1.02** (0.87 to 1.20)	50 per 1,000	**1 more per 1,000** (from 7 fewer to 10 more)
**Mid-term Stroke**											
8396 (7 RCTs)	not serious	not serious	not serious	serious[a]	none	⊕⊕⊕◯ Moderate	110/4207 (2.6%)	85/4189 (2.0%)	**RR 0.79** (0.60 to 1.05)	26 per 1,000	**5 fewer per 1,000** (from 10 fewer to 1 more)
**Mid-term Coronary reintervention**											
10572 (9 RCTs)	not serious	not serious	not serious	not serious	none	⊕⊕⊕⊕ High	98/5294 (1.9%)	146/5278 (2.8%)	**RR 1.49** (1.16 to 1.92)	19 per 1,000	**9 more per 1,000** (from 3 more to 17 more)

CI: confidence interval; **OR:** odds ratio; **RR:** risk ratio

Explanations

a. In Trial sequential analysis, the cumulative curve neither crosses a sequential monitoring boundary nor an invalid boundary before reaching the RIS. Additionally, it does not reach the RIS. Therefore, there may be no difference for the incidence of this outcome between on-pump CABG and off-pump CABG. However, this conclusion still requires more trials to verify.

Figure S11.3 GRADE evidence of profile of long-term outcomes

**Certainty assessment**							**Summary of findings**				
**Participants (studies) Follow-up**	**Risk of bias**	**Inconsistency**	**Indirectness**	**Imprecision**	**Publication bias**	**Overall certainty of evidence**	**Study event rates (%)**		**Relative effect (95% CI)**	**Anticipated absolute effects**	
							**With On-pump**	**With Off-pump**		**Risk with On-pump**	**Risk difference with Off-pump**
**Long-term All-cause mortality**											
10811 (8 RCTs)	not serious	not serious	not serious	serious[b]	none	⊕⊕⊕◯ Moderate	873/5409 (16.1%)	968/5402 (17.9%)	**RR 1.09** (1.01 to 1.17)	161 per 1,000	**15 more per 1,000** (from 2 more to 27 more)
**Long-term Stroke**											
5341 (3 RCTs)	not serious	not serious	not serious	serious[a]	none	⊕⊕⊕◯ Moderate	76/2669 (2.8%)	60/2672 (2.2%)	**OR 0.79** (0.57 to 1.11)	28 per 1,000	**6 fewer per 1,000** (from 12 fewer to 3 more)
**Long-term Coronary reintervention**											
9891 (6 RCTs)	not serious	not serious	not serious	serious[a]	none	⊕⊕⊕◯ Moderate	238/4941 (4.8%)	277/4950 (5.6%)	**RR 1.11** (0.96 to 1.28)	48 per 1,000	**5 more per 1,000** (from 2 fewer to 13 more)

CI: confidence interval; **OR:** odds ratio; **RR:** risk ratio

Explanations

a. In Trial sequential analysis, the cumulative curve neither crosses a sequential monitoring boundary nor an invalid boundary before reaching the RIS. Additionally, it does not reach the RIS. Therefore, there may be no difference for the incidence of this outcome between on-pump CABG and off-pump CABG. However, this conclusion still requires more trials to verify.
b. In Trial sequential analysis, when the cumulative curve reaches the RIS, it crosses the conventionally significant boundary but does not cross sequential monitoring boundary. Therefore, there may be a higher long-term mortality rates associated with off-pump CABG compared to on-pump CABG. This conclusion still requires more trials to verify.
